# Measuring functional connectivity with wearable MEG

**DOI:** 10.1016/j.neuroimage.2021.117815

**Published:** 2021-01-29

**Authors:** Elena Boto, Ryan M. Hill, Molly Rea, Niall Holmes, Zelekha A. Seedat, James Leggett, Vishal Shah, James Osborne, Richard Bowtell, Matthew J. Brookes

**Affiliations:** a Sir Peter Mansfield Imaging Centre, School of Physics and Astronomy, University of Nottingham, University Park, Nottingham, NG7 2RD, United Kingdom; b QuSpin Inc., 331 South 104th Street, Suite 130, Louisville, 80027, CO, USA

**Keywords:** Optically-pumped magnetometer, OPM, Magnetoencephalography, MEG, OPM-MEG, Functional connectivity, Network, Wearable MEG, Amplitude-envelope correlation, AEC

## Abstract

Optically-pumped magnetometers (OPMs) offer the potential for a step change in magnetoencephalography (MEG) enabling wearable systems that provide improved data quality, accommodate any subject group, allow data capture during movement and potentially reduce cost. However, OPM-MEG is a nascent technology and, to realise its potential, it must be shown to facilitate key neuroscientific measurements, such as the characterisation of brain networks. Networks, and the connectivities that underlie them, have become a core area of neuroscientific investigation, and their importance is underscored by many demonstrations of their disruption in brain disorders. Consequently, a demonstration of network measurements using OPM-MEG would be a significant step forward. Here, we aimed to show that a wearable 50-channel OPM-MEG system enables characterisation of the electrophysiological connectome. To this end, we measured connectivity in the resting state and during a visuo-motor task, using both OPM-MEG and a state-of-the-art 275-channel cryogenic MEG device. Our results show that resting-state connectome matrices from OPM and cryogenic systems exhibit a high degree of similarity, with correlation values *>*70%. In addition, in task data, similar differences in connectivity between individuals (scanned multiple times) were observed in cryogenic and OPM-MEG data, again demonstrating the fidelity of the OPM-MEG device. This is the first demonstration of network connectivity measured using OPM-MEG, and results add weight to the argument that OPMs will ultimately supersede cryogenic sensors for MEG measurement.

## 1. Introduction

Since its inception, functional neuroimaging has made many important contributions to our understanding of brain function, and one of the most significant is the discovery of brain networks. A network is found when a statistical relationship between neuroimaging signals, derived from two or more spatially separate brain regions, is shown to exist. Such a relationship is termed functional connectivity. The first measurements of functional connectivity used functional magnetic resonance imaging (fMRI; Biswal et al. (1995)) to measure correlation between blood-oxygenated-level-dependent (BOLD) time courses from left and right motor cortex, in the absence of a task (in the so-called “resting state”). Following this, many fMRI studies (e.g. (Beckmann et al., 2005; Fox and Raichle, 2007; Smith et al., 2009)) focused on identifying other resting-state networks (RSNs); some associated with sensory processing (e.g. auditory or visual networks) and others with attention and cognition (e.g. the default-mode and dorsal-attention networks). Raichle (2009) described this era of functional imaging as a «paradigm shift». Indeed, study of RSNs offers a powerful means to investigate healthy function, and dysfunction in a wide range of disorders, including schizophrenia, depression, anxiety and dementia (Menon, 2011).

Most functional connectivity studies have been based on fMRI. However, the BOLD signal is an indirect metric of function, based on haemodynamics, which leads to significant disadvantages: for example, Bright et al. (2020) showed an overlap between neural and vascular network components and this makes the interpretation of fMRI networks challenging without first understanding network-specific vascular architecture. In addition, whilst fMRI exhibits exquisite spatial resolution (~1 mm accuracy), it has limited temporal resolution due to the latency and longevity of the haemodynamic response. This means that the time scale of a functional connectivity measurement is limited, and so it is challenging to probe the formation and dissolution of functional networks on a time scale relevant to cognition (Hutchison et al., 2013). For these reasons, a move towards electrophysiological imaging techniques (which exhibit significantly better temporal resolution) for the characterisation of network connectivity is important.

Magnetoencephalography (MEG; Cohen (1972)) measures the magnetic fields generated outside the head by current flow through neuronal assemblies in the brain. In this way, it offers a means to bypass haemodynamics and infer directly the electrophysiological connectome. MEG data (like all electrophysiological data) are dominated by “neural oscillations” (rhythmic electrical activity synchronised across neurons) in the 1–200 Hz frequency range. Emerging evidence suggests that these oscillations mediate (in part) network formation and consequently their measurement offers an exciting means to probe network coupling (Engel et al., 2013). MEG has been used successfully to measure functional connectivity in many studies (e.g. (Baker et al., 2014; Brookes et al., 2011b, 2012; Gross et al., 2001; Hipp et al., 2012; Luckhoo et al., 2012; O’Neill et al., 2015)). Networks similar to those seen with fMRI have been found and the excellent temporal resolution enables measurement of dynamic connectivity. Indeed, recent studies show that canonical networks modulate on the time scale of seconds (O’Neill et al., 2015) and even milliseconds (Baker et al., 2014). More recently, Seedat et al. (2020) suggested the involvement of extremely short (e.g. ~300 ms) punctate events in driving canonical network connectivity, further underscoring the importance of temporal precision.

A combination of high spatial and temporal accuracy means that MEG offers arguably the best means to measure functional connectivity. However, existing MEG systems have huge limitations: the sensors that form the basic building block of MEG systems (superconducting quantum interference devices; SQUIDs) operate at cryogenic temperatures. These sensors must therefore be fixed in position within a cryogenic dewar, making systems large, cumbersome, and “one-size-fits-all” – i.e. they cannot adapt to different head shapes or sizes. This results in inhomogeneous and sometimes poor brain coverage (particularly in infants). Even if the head is well fitted to a MEG system, the gap between the scalp and the sensors that is needed for thermal insulation reduces sensitivity (according to an inverse square law). The fixed nature of the sensors also means that any head movement during data acquisition can significantly reduce data quality. For this reason, ideally, subjects should remain extremely still, which makes the environment poorly tolerated by many groups, again including children. In recent years, a number of algorithms have become available that are able to “correct” for head movements inside a MEG helmet. However, the degree of movement remains limited (by the size of the helmet itself) and no algorithm can correct for changing signal-to-noise ratio (SNR) in specific brain regions as the brain gets closer to, or further from sensors. For this reason, even with movement compensation algorithms, motion (particular at large scale) remains problematic. Finally, the cryogenic infrastructure and complex electronics make systems expensive. These factors have, to date, limited the uptake of MEG, and if MEG-based connectome measures are to realise their potential for neuroscientific discovery and clinical translation, then new types of MEG technology will be required.

Recent advances in quantum technology have led to the development of a new type of magnetic field sensor. Optically-pumped magnetometers (OPMs) offer measurement of magnetic field with a similar sensitivity to the cryogenic sensors used in conventional MEG, however they do not require cooling. Furthermore, they are small and lightweight. This has led a number of groups to begin to fabricate OPM-based MEG devices. Suitability of OPMs to capture neuromagnetic signals has been well documented (e.g. (Barry et al., 2019; Borna et al., 2020; Boto et al., 2017; Iivanainen et al., 2019; Johnson et al., 2013; Kamada et al., 2015; Kim et al., 2014; Roberts et al., 2019; Sander et al., 2012; Tierney et al., 2018; Xia et al., 2006) and more recently their lightweight nature has been exploited to develop “wearable” systems in which (if background fields are controlled appropriately) subjects can move freely during data acquisition (Boto et al., 2018). OPM arrays are beginning to be developed with up to 50 sensors surrounding the head (Hill et al., 2020) and there is a growing argument that these devices – which also have the potential to be cheaper than conventional MEG – will ultimately supersede the current generation of scanners. However, OPM-MEG remains a nascent technology and if the functional neuroimaging field is to gain confidence in it, OPM-based systems must be able to do everything a SQUID system can do. Given the importance of functional connectivity, demonstration of its measurement with OPM systems is a vital step.

There are a number of reasons why functional connectivity is a challenge for OPM-MEG. Firstly, most OPM experimental demonstrations have targeted specific brain regions due to a relatively low sensor count. Given that networks are distributed across the whole brain, coverage that rivals conventional MEG is required. Secondly, networks are spatially-specific and their measurement relies on the ability to project magnetic field data into source space (a process termed source localisation; (Schoffelen and Gross, 2009)). Consequently, if an OPM system is to characterise networks then it must offer the ability to accurately localise large numbers of sources, across the whole cortex, with high spatial accuracy. In reality this means achieving equivalent reconstruction accuracy with around 50 OPMs, rather than ~300 cryogenic sensors. Finally, functional connectivity is heavily reliant on high quality data since, unlike task-based studies where data can be averaged over many trials (and thus artefacts will often be averaged out) functional connectivity (particularly in the resting state) must be measured using unaveraged data. This latter point is amplified since unlike conventional MEG systems which often rely on a gradiometer formulation to reduce environmental interference, commercially-available OPMs are naturally formed as magnetometers. This, at least in principle, increases the effect of external interference, both from environmental (e.g. lab equipment) and biological (e.g. the heart) sources. Such interference can artificially inflate or reduce connectivity estimates, especially in unaveraged data.

In this paper, we aim to test whether a 50-channel OPM-MEG system can successfully measure the electrophysiological functional connectome. To this end, we measure connectivity during a visuo-motor task, and in the resting state. In both cases we compare quantitatively our OPM findings to equivalent measures generated using a 275-channel cryogenic system.

## 2. Methods

### 2.1. OPM-MEG system

**Fig. 1.**
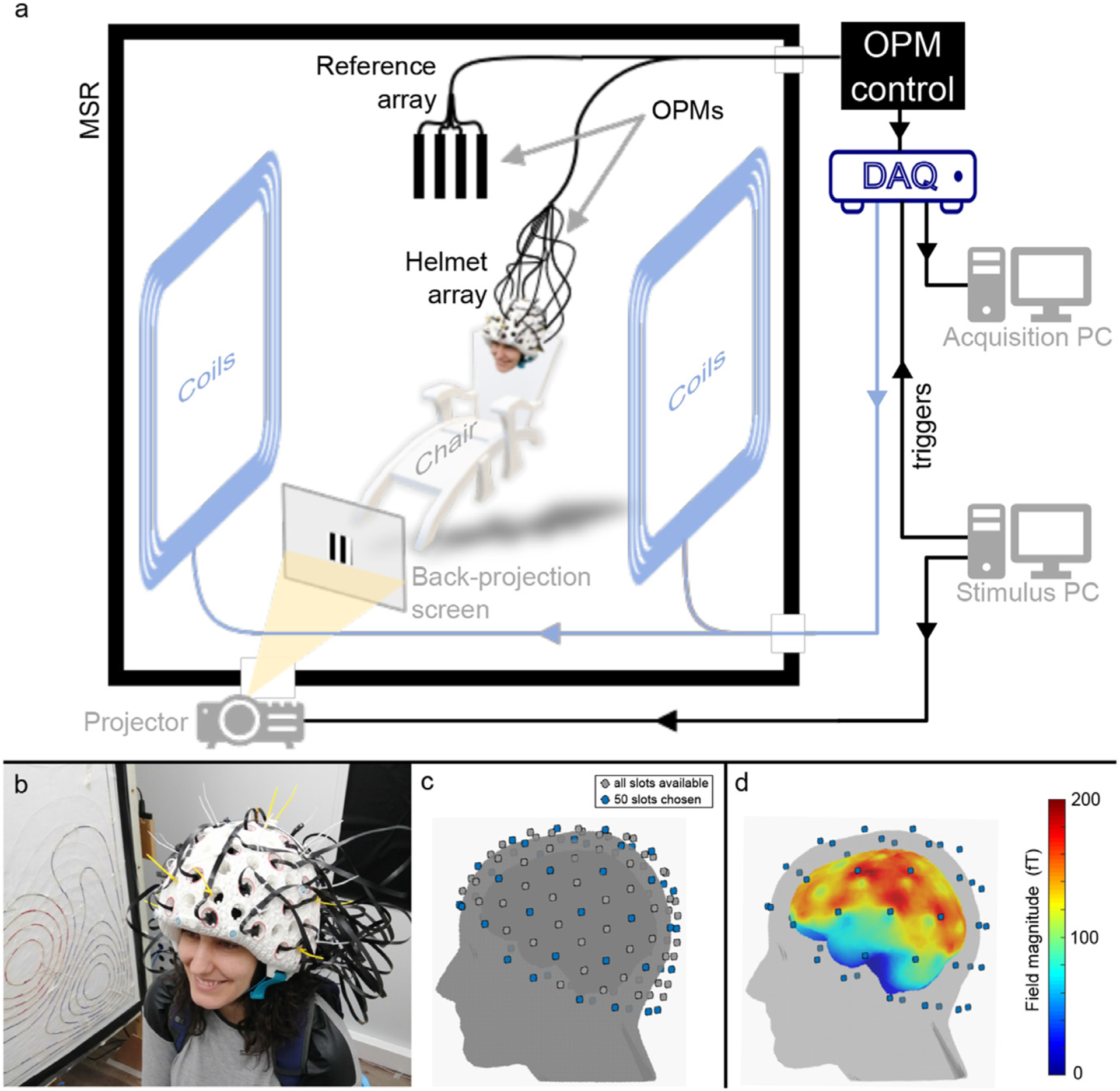
The OPM-MEG system. a) Schematic of the OPM-MEG suite. b) Photograph of subject wearing an additively-manufactured helmet with 50 OPM sensors mounted within it. c) Digitised head surface for an example participant, showing the 133 slots available in the helmet (grey) and the 50 chosen for this study (blue). Note that OPMs were made sensitive to the field in the radial direction only. d) Cortical coverage achieved by the selected 50 OPM locations: the norm of the forward fields across all sensors is plotted at each vertex of the brain surface.

The wearable OPM-MEG device used in this study has been developed at the Sir Peter Mansfield Imaging centre, University of Nottingham, and was described recently in Hill et al. (2020). A schematic representation of the system is shown in Fig. 1a: the OPM-MEG suite contains a magnetically-shielded room (MSR), the design of which has been optimised for OPM operation (MuRoom, Magnetic Shields Ltd. Kent, UK). This MSR provides a remnant magnetic field magnitude *<*2 nT and *<*2 nT/m magnetic field gradient following a demagnetisation procedure (Altarev et al., 2015). These fields are significantly lower than those found in MSRs that do not feature demagnetisation coils.

Inside the MSR, the participant sits in a non-magnetic chair, and wears an additively-manufactured rigid helmet in which 50 OPMs (second generation zero-field magnetometers, QuSpin Inc., (Osborne et al., 2018)) are mounted (see photograph in Fig. 1b). The helmet contains a total of 133 possible slots where OPMs can be placed (grey dots in Fig. 1c), and for this study, we chose 50 locations to provide whole-head coverage (blue dots). The OPMs themselves were configured to measure the radial component of magnetic field. Fig. 1d shows the cortical coverage achieved with 50 radial OPMs placed in the slots shown in blue: for each vertex of the brain surface, the colour represents the norm of the field (across all sensors) produced by a current dipole located at that vertex. (Dipoles were oriented in two orthogonal tangential directions, and the result is taken to be the average of the two lead field norms.) Note that reasonable coverage of the whole cortex is achievable, although sensitivity to the temporal pole is somewhat diminished (a problem also found in conventional MEG devices).

Further reduction of background magnetic fields inside the MSR is achieved using a set of bi-planar coils (Holmes et al., 2018) positioned either side of the participant. These coils are coupled to an OPM reference array placed behind the subject’s head. The remnant static magnetic field and its (linear) spatial variation inside the MSR is estimated by the reference array, and an equal and opposite field applied by the coils in order to effect field cancellation. This reduces the remnant field (typically to *<*1 nT), better enabling OPM-MEG operation by minimising any artefact caused by the subject moving their head (and consequently the sensors) through the background field.

The OPMs themselves have been described previously and a complete description will not be repeated here (see e.g. Tierney et al. (2019) or Boto et al. (2020) for a review). Note that they are controlled via a computer located outside the MSR, and analogue output signals proportional to local magnetic field, are fed into a National Instruments (Austin, TX, USA) Digital Acquisition unit (DAQ), digitised, and recorded. A separate computer is coupled to stimuli delivery systems and sends triggers to the DAQ which are synchronously recorded with the MEG data.

### 2.2. OPM co-registration

In order to enable source localisation, accurate knowledge of the OPM sensor locations and orientations relative to the brain is required. This was provided by a 3-dimensional optical imaging system (structure IO camera (Occipital Inc., San Francisco, CA, USA) coupled to an Apple iPad, operating with Skanect Pro software) and an anatomical MRI scan (Hill et al., 2020; Homölle and Oostenveld, 2019; Zetter et al., 2019). The anatomical MRI was recorded using a 3 T Philips Ingenia MRI system, running an MPRAGE sequence, at an isotropic spatial resolution of 1 mm. The locations and orientations of the sensor casings with respect to the helmet are known *a-priori* from the additive manufacturing process, and the co-registration procedure was used to map the helmet on to the head. This was done in two stages. First, 6 coloured markers were placed at known locations on the helmet, with a further 4 on the participant’s face. The camera, coupled with a colour-thresholding algorithm, was used to map the relative locations of these markers, allowing mapping of the helmet to the face. Following this, the helmet was removed and the participant was asked to wear a swimming cap (to flatten their hair). A second digitisation was then acquired measuring the positions of the markers on the face, relative to the head surface. The head surface was then fitted to the equivalent surface extracted from the anatomical MRI scan. Combining two transforms (helmet-to-head and head-to-MRI) we were able to effect a complete co-registration of sensor casing to brain anatomy. The location of the sensitive cell within the OPM casing was accounted for and we assumed that the sensitive axis was radial, and parallel to the external sensor housing.

### 2.3. Task-based connectivity experiment

The data used for our task-based connectivity demonstration have been previously reported by Hill et al. (2020).

#### 2.3.1. Paradigm and data acquisition

Two subjects undertook a visuo-motor task. The task comprised presentation of a centrally-presented, inward-moving, maximum-contrast circular grating (Hoogenboom et al., 2006; Iivanainen et al., 2019), which is known to increase gamma oscillations in the visual cortex. Whilst the visual stimulus was on the screen, participants were asked to perform continuous abductions of their right index finger; a task known to modulate beta oscillations in sensorimotor cortex. The grating was presented for either 1.6, 1.7 or 1.9 s, depending on the trial. Each trial ended with a 3-s baseline period, and 100 trials were recorded.

Both participants were scanned six times in the OPM-MEG system and six times in a cryogenic MEG instrument (CTF, Coquitlam, BC, Canada). The study was approved by the University of Nottingham Medical School Research Ethics committee.

OPM data were acquired using a sampling frequency of 1,200 Hz. 42 and 49 OPM sensors were available for participants 1 and 2, respectively. The visual stimulus was back projected onto a screen placed ~85 cm in front the participant’s head. A separate co-registration procedure was performed after each experiment.

Cryogenic MEG data were acquired using a 275-channel CTF system, operating in 3rd-order gradiometer configuration, at a sampling frequency of 600 Hz. The stimulus was presented on a back-projection screen placed 95 cm in front of the participant (the stimulus was matched for visual angle between the two scanner types). Three head-position indicator (HPI) coils were placed on the participant’s head at three fiducial locations (nasion, left and right pre-auricular points). Continuous tracking of the head was achieved via these coils, which were periodically energised during MEG data acquisition. A 3D digitiser (Polhemus) was used to measure the locations of the fiducial markers relative to the head surface, prior to each experiment. By matching the participant’s digitised head surface with the equivalent surface extracted from their anatomical MRI, co-registration of the fiducial markers, and consequently the MEG sensor geometry, to the individual’s brain anatomy was achieved.

#### 2.3.2. Task-based data analysis

All MEG data (OPM and cryogenic) were band-pass filtered between 8–80 Hz and epoched into trials (−3.5 s to + 3.0 s relative to time zero, which was taken as stimulus onset; giving a 6.5-s trial length). A “bad” trial was removed (completely; i.e. all channels removed) in cases where the standard deviation of the signal (in any single channel) was greater than 3 times the average standard deviation across all trials (for the same channel). Data were then concatenated into a single signal per channel.

Both cryogenic and OPM data were analysed in the same way: functional connectivity was calculated between 78 discrete cortical regions, defined based on the automated anatomical labelling (AAL) atlas (Tzourio-Mazoyer et al., 2002). A scalar beamformer was used to obtain a single electrophysiological time course representative of each region (i.e. a ‘virtual electrode’ placed at the centre of mass of each region (Hillebrand et al., 2016). The data covariance matrix was computed in the 8–80 Hz frequency range for a time window spanning the whole experiment. Regularisation was not applied, to maximise spatial resolution (Brookes et al., 2008). The forward model was based on a single sphere for the OPM system and multiple local spheres for the cryogenic system.

After beamforming, regional signals were frequency-filtered to the alpha (8–13 Hz), beta (13–30 Hz), and gamma (52–80 Hz) frequency bands, and epoched into trials. Pairwise orthogonalization (Brookes et al., 2012; Hipp et al., 2012) was used to mitigate the problem of signal leakage between AAL regions (itself a result of the ill-posed MEG inverse problem). Following this, the absolute value of the Hilbert transform of the frequency-filtered data was computed to generate the amplitude envelope of oscillatory signals, which was then down-sampled to 10 Hz. (Note down sampling in this way has been used in previous connectivity studies (Brookes et al., 2011a); whilst a lower frequency cut-off is typically used for resting-state data we employed 10 Hz to ensure that task-induced dynamics in the envelope were maintained.) Pearson correlation was calculated between amplitude signals for all possible AAL region pairs and averaged over trials. For each participant, each experiment and each frequency band, this procedure resulted in a single 78 × 78 matrix representing whole-brain connectivity. Finally, connectivity matrices were averaged across experimental runs.

OPM and cryogenic results in the beta band were compared. (Note we chose the beta band because this range has been shown to provide robust brain network measurements (e.g. Hunt et al. (2016)). Comparisons were made in two ways:

##### Connectome repeatability (correlation):

For each subject, we have 12 connectivity matrices (6 OPM and 6 cryogenic). Our aim was to assess how similar these matrices are; that is, “how repeatable is the whole-brain connectome across experimental runs?” We wished to compute this repeatability within/between scanner types (i.e. OPM-to-OPM; cryogenic-to-cryogenic and OPM-to-cryogenic) and within/between subjects (i.e. subject 1 – to – subject 1; subject 2 – to – subject 2; subject 1 – to – subject 2). To this end, we first vectorised the matrices from all experimental runs. The 6 OPM runs and 6 cryogenic runs (for the same subject) were paired (all 36 possible pairings used) and Pearson correlation between the vectorised connectivity matrices was calculated. This results in 36 values of correlation which were averaged. In this way, we obtained a within-subject correlation between OPM and cryogenic connectivity matrices. This comparison was repeated for both subjects. We then performed the equivalent calculation between subjects (e.g. correlating connectivity from subject 1′s cryogenic data and subject 2′s OPM data, and *vice versa*). Combined, this gave measures of repeatability within and between subjects, across scanner platforms. In addition, we also calculated repeatability within scanner platform: i.e. OPM-to-OPM and cryogenic-to-cryogenic. Here, pairing separate experimental runs (e.g. run 1 to run 2; run 1 to run 3 etc.) allowed 15 values of correlation to be derived within a subject and 36 values of correlation to be derived between subjects. This analysis allowed the calculation of measures of repeatability within and between subjects, but this time within scanner platforms. Bringing together all these measures allowed us not only to assess the repeatability of connectivity matrices, but also subject specificity, and sensitivity to scanner platform. We hypothesised that there would be individual differences in the connectivity matrices between subject 1 and subject 2, and that these differences would be maintained across the two scanner types (i.e. colloquially, “the scanner would know who it was scanning’”). Consequently, we expected that correlation values would be higher within subject than between subject.

##### Connectivity strength:

We calculated the linear sum of elements within each connectivity matrix, in one direction; this resulted in 78 regional values of “connectivity strength” (i.e. for each of the AAL regions, this metric represents the strength of the connection between that region and all other regions in the AAL atlas). These values were normalised within each run, by dividing by the maximum value across all regions. Connectivity strength was separately calculated for each subject, MEG system, and experimental run. Here, we wished to probe whether individual differences in connectivity strength were maintained across the two MEG systems. To this end, for each of the 78 regions, a *t*-test (see also Supplementary Material) was used to determine the statistical significance of differences between subjects. These calculations were performed for each scanner type separately (i.e. for a single scanner, and a single region, we tested whether the 6 connectivity strength measures from subject 1 were significantly different to the equivalent 6 connectivity strength measures from subject 2). Multiple comparisons (across the 78 regions) were controlled using the Benjamini-Hochberg procedure (Benjamini and Hochberg, 1995). We hypothesised that any regions where a significant between-subject difference occurred, would be matched across scanner types.

### 2.4. Resting-state connectivity experiment

#### 2.4.1. Paradigm and data acquisition

Seven subjects (2 females, mean age 26 ± 4 years) took part in the resting-state study. All participants gave written informed consent, and the study was approved by the University of Nottingham Medical School Research Ethics Committee.

Seven minutes of eyes-open, resting-state MEG data were acquired using the wearable OPM-MEG system at a sampling frequency of 1,200 Hz. Participants were asked to fixate on a small red cross which was centrally positioned on a grey background on the back-projection screen. Apart from this, they were simply asked to relax and do nothing. Participants were free to move during the recording, but they were not encouraged to do so. Co-registration was performed (as described above) at the end of each experiment, and individual anatomical MRIs were available for all participants.

For comparison, we employed eyes-open resting-state data which had been acquired previously, in 63 subjects as part of the United Kingdom MEG Partnership (UKMP; (Hunt et al., 2016)) programme. These data were all acquired using the same 275-channel cryogenic MEG system used in our task-based study. The system was operated in 3rd-order synthetic gradiometer configuration, and data were acquired at a sampling frequency of 1,200 Hz. The paradigm comprised a 5-min recording during which participants focused on a small, centrally-positioned red circle, back-projected onto a screen. Head position monitoring was facilitated via three head-position indicator coils which were energised during the scan. Co-registration of MEG sensor geometry to individual brain anatomy was achieved via head shape digitisation, equivalent to that described above for our task-based study.

#### 2.4.2. Resting-state data analysis

Data were band-pass filtered between 8–80 Hz, and bad channels were discarded based on visual inspection. This meant there were OPM data from 49, 47, 47, 48, 47, 48 and 45 channels for subjects 1–7, respectively.

OPM and cryogenic data were processed in the same way. A scalar beamformer was employed to reconstruct a representative signal at the centre of mass of each of the 78 cortical AAL regions. Data covariance was computed in the 8–80 Hz frequency band and within a time window encompassing the complete resting-state recording. Regularisation was not used (see also Appendix). Following this, regional signals were frequency-filtered into the alpha (8–13 Hz) and beta (13–30 Hz) bands, and pairwise orthogonalisation used to mitigate signal leakage. The absolute value of the analytic signal was computed for each regional time course, to generate the amplitude envelope of oscillatory signals, which was then down-sampled to 5 Hz (the lower frequency cut-off was used because we expected envelopes to fluctuate more slowly in the resting state). Pearson correlation was calculated between envelopes for each region pair. For each participant and frequency band, this resulted in a single connectivity matrix representing whole-brain connectivity. Group connectivity matrices were computed by averaging across subjects.

We aimed to show that OPM-derived connectivity was similar to connectivity derived using a cryogenic instrument and to this end we exploited the large UKMP dataset. We randomly grouped the cryogenic-derived connectivity matrices from our 63 subjects into 9 groups, with 7 subjects per group. For each frequency band, we computed a group average connectivity matrix. This resulted in 9 matrices derived from cryogenic data which we could compare with the single average (also of 7 subjects) derived from our OPM system.

Quantitative analysis of the similarity between OPM- and cryogenic-derived connectivity matrices was made in two ways:

First we aimed to test whether the OPM-to-cryogenic correlation was above chance. To test this, we first vectorised connectivity matrices. For both frequency bands, we derived correlations between the OPM group average, and each of the 9 cryogenic average matrices, resulting in 9 values of correlation which were averaged. Following this, to provide a statistical value, we used a Monte-Carlo approach. Starting with the OPM connectome matrix, we used a phase randomisation method (Hunt et al., 2016; O’Neill et al., 2015) to produce a set of “pseudo-matrices”: this method has been described in full in a previous paper (Tewarie et al., 2016). Briefly, the real connectome matrix is decomposed into its constituent eigenvalues and eigenvectors. The eigenvectors are then phase randomised (Prichard and Theiler, 1994), and the matrix is reconstructed. The result is a new matrix with similar structure to the real matrix, but critically not representative of real brain connectivity. We made 10,000 pseudo-matrices and correlated each with the 9 cryogenic-derived (real) connectome matrices. This resulted in 90,000 values of correlation which was used as a null distribution. In order to test whether our real OPM-derived connectivity was correlated beyond chance with cryogenic-derived connectivity, we compared the 9 real correlation values to the null distribution, setting a significance threshold of 1%.

Second, we aimed to test whether the OPM-to-cryogenic connectivity was similar to cryogenic-to-cryogenic connectome correlation. To this end, for each frequency band we measured correlation between all possible pairs of cryogenic-derived connectivity matrices (i.e. group 1 to group 2; group 1 to group 3 etc.). This yields a total of 36 correlation values showing how different randomly-selected groups of individuals compare in terms of their whole-brain (cryogenic-derived) connectome. We reasoned that if OPM-derived connectivity was different to cryogenic-derived connectivity, then we would expect our 9 values of OPM-to-cryogenic correlation to fall outside the range of 36 values obtained when considering cryogenic-to-cryogenic correlation.

## 3. Results

### 3.1. Task-based connectivity

**Fig. 2.**
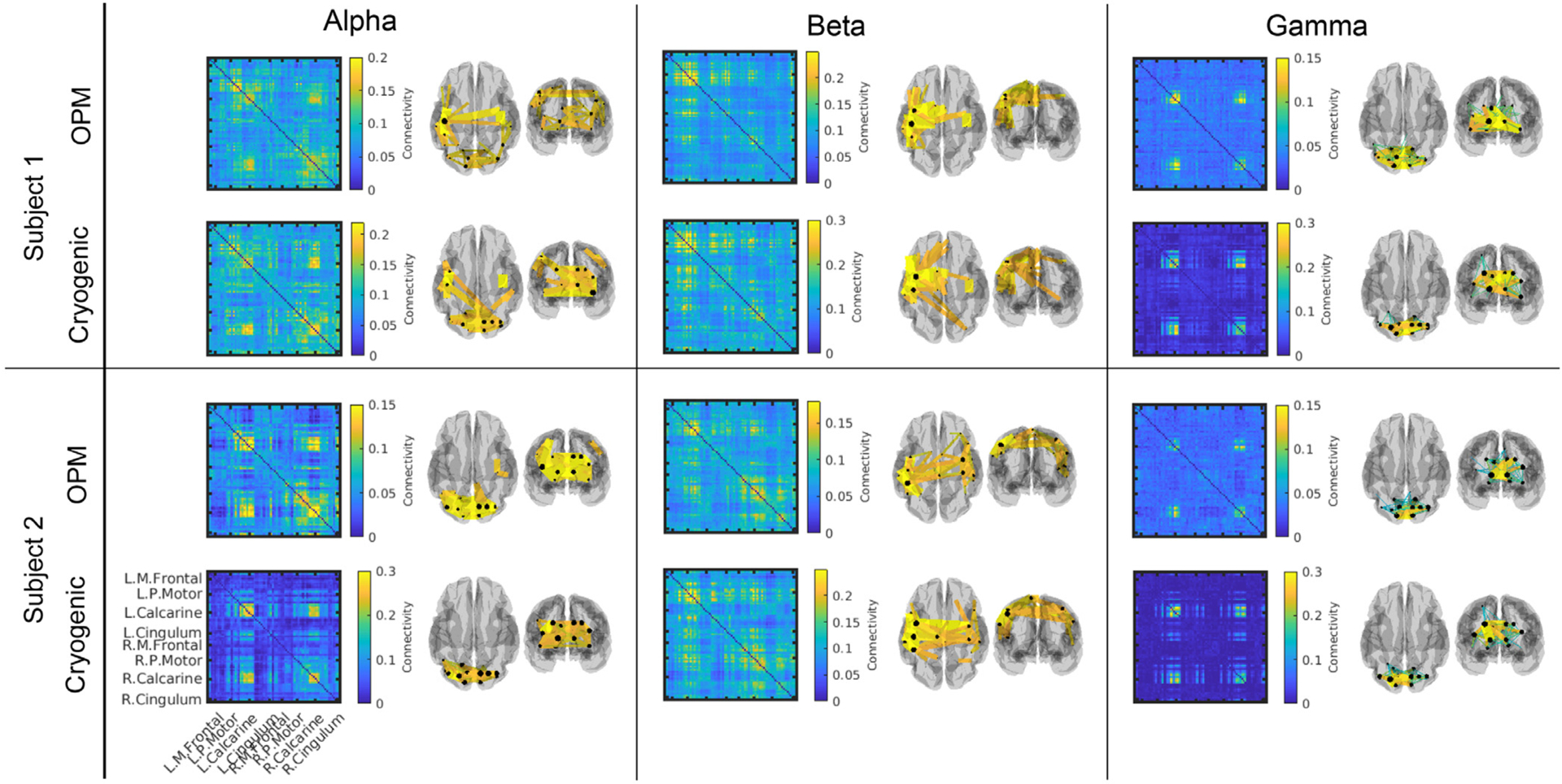
Task-based functional connectivity matrices. Average connectivity matrices (across 6 runs) in the alpha (left), beta (middle) and gamma (right) bands for participants 1 (top) and 2 (bottom). For each participant, both OPM-derived (top) and cryogenic-derived (bottom) matrices are shown. Colour bars show connectivity (i.e. Pearson correlation between amplitude envelope) values. Alongside the matrices, the 3D brains show the 50 connections with the highest connectivity values.

Fig. 2 shows results from our task-based connectivity study. For each participant, connectivity matrices obtained from OPM and cryogenic data, averaged across six experimental runs, are shown. The left panel shows results in the alpha band, the middle panel shows beta band and the right panel shows gamma band. Colour indicates connectivity value. The inset 3D brain plots show the 50 connections between AAL regions with the highest connectivity values (represented by the coloured lines). Clear differences in network structure can be seen between the three bands: the alpha-connectome is predominantly occipital, although subject 1 shows some parietal connections; the beta band shows primarily parietal (bilateral sensorimotor) connections. Anecdotally, we note a more unilateral network in subject 1 and a bilateral network in subject 2. The gamma-band is dominated by occipital connections. Similarities between OPM- and cryogenic-derived matrices are clear, a good example being the agreement on inter-individual differences that are shown in the beta band. This will be formalised below.

An interesting observation from Fig. 2 is that, in the beta band, the OPM-derived connectivity values are lower than the cryogenic-derived values. Specifically, averaging values over the connectivity matrices and computing standard deviation across experimental runs, we found that mean connectivity was 0.10 ± 0.01 (OPM) compared to 0.13 ± 0.02 (cryogenic), for subject 1 and 0.08 ± 0.01 (OPM) compared to 0.11 ± 0.01 (cryogenic) for subject 2. This observation was less marked in the alpha band where mean connectivity was 0.09 ± 0.01 (OPM) compared to 0.10 ± 0.02 (cryogenic), for subject 1 and 0.06 ± 0.01 (OPM) compared to 0.06 ± 0.01 (cryogenic) for subject 2. Conversely in the gamma band, OPM connectivity values were slightly higher: 0.04 ± 0.01 (OPM) compared to 0.03 ± 0.01 (cryogenic), for subject 1 and 0.04 ± 0.01 (OPM) compared to 0.02 ± 0.01 (cryogenic) for subject 2. This observation will be addressed further in the resting state results, and in the Discussion.

**Fig. 3.**
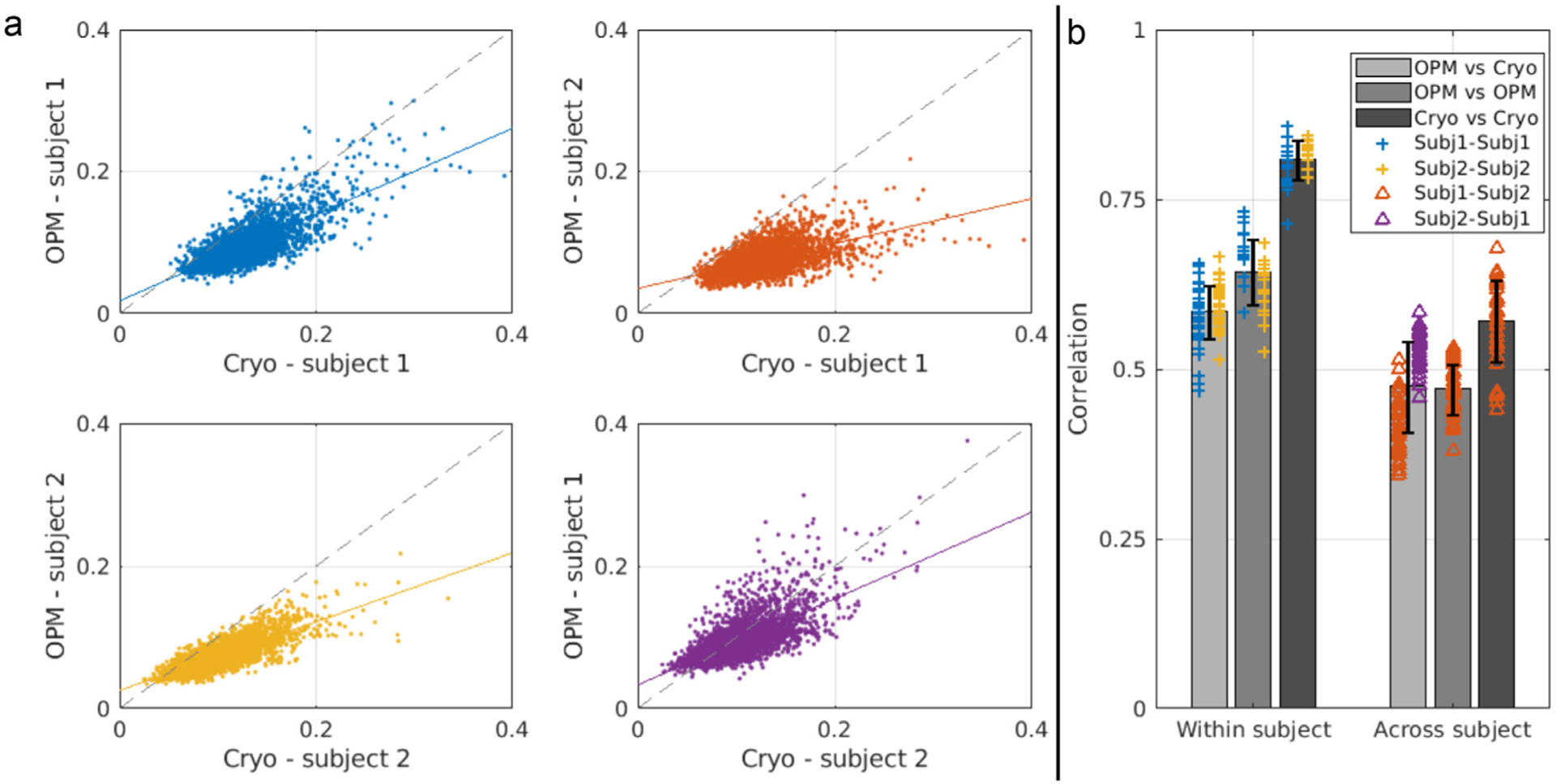
Cryogenic vs OPM connectivity in the beta band. a) Scatter plots showing connectivity values derived from cryogenic data plotted against connectivity values derived from OPM data (each dot depicts a measured connection). Left column shows within-subject correlation for subject 1 (top) and subject 2 (bottom). Right column corresponds to between-subject correlation. b) Bar plot showing the mean within- and between-subject correlation of connectome matrices. Connectome repeatability is calculated in three ways; cryogenic-to-cryogenic (dark grey; here we compare connectome matrices taken using the cryogenic system in separate runs); OPM-to-OPM (middle grey; comparing matrices taken using the OPM system in separate runs); and OPM-to-cryogenic (light grey; comparing matrices derived using the OPM system to matrices derived using the cryogenic system). Error bar corresponds to standard deviation across the 15 or 36 comparisons. Crosses and triangles indicate individual values from a single calculation of correlation between two matrices – i.e. all raw data are shown.

Fig. 3 probes the similarity of connectome matrices, within- and between-subjects, in OPM and cryogenic recordings. Comparison is made in the beta band only. In panel a, scatter plots show OPM-derived connectivity values for all region pairs (*y*-axis), plotted against the equivalent cryogenic-derived connectivity values (*x*-axis). I.e. each point on the graph represents a measured connection, and assuming OPMs and cryogenic sensors measure the same connectivities, in the same subject, we would expect to see a linear relationship. Plots on the left show a within-subject comparison: the top scatter plot (blue) corresponds to subject 1 and the bottom plot (yellow) corresponds to subject 2. The scatter plots on the right compare connectivity matrices between subjects (i.e. subject 1 – cryogenic vs subject 2 – OPM, and *vice versa*). Note that to generate these plots, we averaged connectome matrices over all 6 runs in both MEG systems. A line of best fit is added, and the dotted line shows ‘*y* = *x*’. Note that a clear linear relationship is observed, demonstrating that OPM- and cryogenic-derived connectivity matrices are similar. Also within-subject correlation (left-hand scatter plots) appears tighter than between-subject correlation (right-hand scatter plots). Finally note that the linear trend is not distributed around *y* = *x*, again suggesting lower connectivity values measured via OPMs.

The bar chart in Fig. 3b shows within- and between-subject repeatability (correlation) of connectome matrices (i.e. these values show how repeatable task-based connectivity is between experimental runs; in other words they show the strength of the correlation in the scatter plots in panel a). Correlation values were split into three groups: OPM-to-OPM; cryogenic-to-cryogenic and OPM-to-cryogenic. The bar chart shows the mean value (across subjects and runs) and the error bars depict corresponding standard deviations. In addition, individual data points relating to all possible comparisons are shown: specifically, blue crosses show subject 1 – to – subject 1; yellow crosses show subject 2 – to – subject 2; red triangles show subject 1 – to – subject 2 and purple triangles show subject 2 – to – subject 1. Importantly these correlation values are relatively high in all cases, but the within-subject values are consistently higher than the between-subject correlations. This is an important point because it suggests that differences in the con nectome matrix between the two subjects are maintained across experimental runs; or more colloquially, we can identify which subject was being scanned based on the connectivity matrix. Repeatability of connectivity was higher for within-subject measures regardless of whether this was measured from data acquired on the OPM system, cryogenic system, or both, demonstrating the robustness of the finding. Interestingly, repeatability was highest for the cryogenic system (i.e. dark grey bars show the highest correlation when measured within and between subjects). This is likely due to the inhomogeneous coverage afforded by a cryogenic system and will be addressed further in the Discussion.

**Fig. 4.**
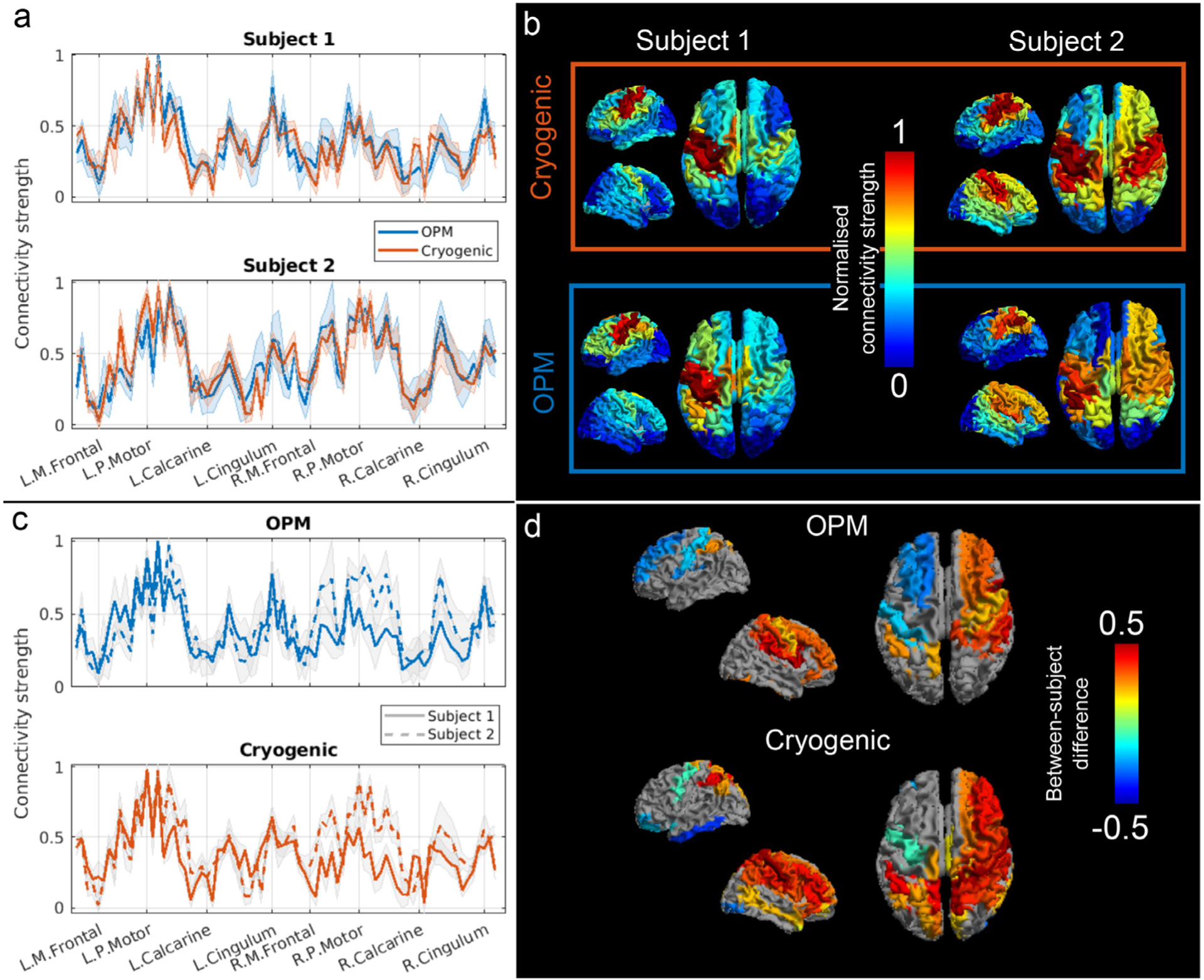
Connectivity strength in the beta band. a) Normalised connectivity strength recorded using cryogenic- (red) and OPM- (blue) derived data. Values are plotted for all 78 AAL regions, for participants 1 (top) and 2 (bottom). The shaded area represents standard deviation across 6 runs. Note the similarities between cryogenic and OPM plots. b) Normalised connectivity strength plotted on the brain surface for both subjects and both systems. c) Same as (a) but grouped by scanner type: normalised connectivity strength recorded using cryogenic- (bottom) and OPM- (top) derived data for participants 1 (solid line) and 2 (dashed line). d) Brain areas showing significant difference between participants (grey indicates no significant difference). Note both systems highlight similar regions.

Results of our connectivity strength analysis are shown in Fig. 4. Panel a shows line plots depicting normalised connectivity strength from the cryogenic- (red) and OPM-derived (blue) beta-band connectome, for subject 1 (top) and 2 (bottom). On the *x*-axis each of the AAL regions are represented. Thick lines correspond to the average connectivity strength and shaded areas represent standard deviation, across all 6 runs. There are clear similarities between the cryogenic- and OPM-derived values with the regions with highest values corresponding to sensorimotor areas (which is to be expected given the task). This is better visualised in Fig. 4b, where the normalised connectivity strength is plotted on a 3D brain. Cryogenic results are at the top, OPM results at the bottom, left panels correspond to subject 1 and right panels to subject 2. For each subject, both cryogenic and OPM data yield very similar connectivity strength patterns. In agreement with Fig. 2, we see that subject 1 exhibits a more unilateral beta-band connectome, as opposed to subject 2, in which a clear bilateral network can be observed. Panel c shows the same data as in panel a but grouped by scanner type: OPM at the top, cryogenic at the bottom. In both plots, subject 1 is represented with a solid line and subject 2 with dashed line. Here, the difference in connectivity strength between both participants around the right sensorimotor areas can be seen clearly. Finally, Fig. 4d shows brain regions whose connectivity strength differed significantly (*p <* 0.05, unpaired *t*-test with 6 degrees of freedom, corrected for multiple comparisons) between subjects. Both cryogenic (bottom) and OPM (top) data highlight similar regions – in particular right sensorimotor cortices and pre-motor areas stretching forward to the frontal lobe. This result formalises the finding in the bar chart in Fig. 3, by demonstrating why within-subject correlation is higher than between-subject correlation, for both OPM and cryogenic systems.

### 3.2. Resting-state connectivity

**Fig. 5.**
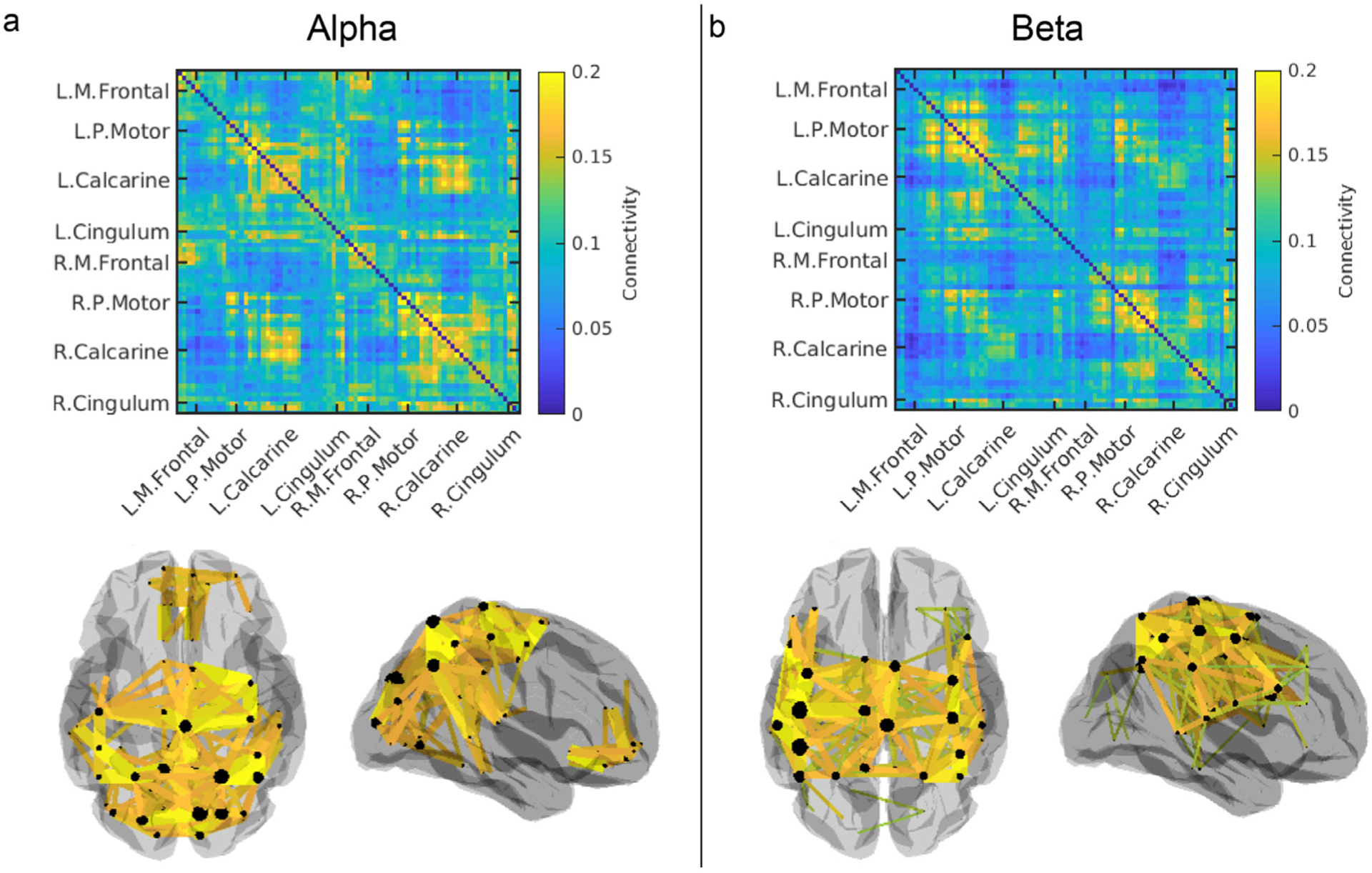
Resting-state connectivity plots derived from OPM data. Alpha- (a) and beta- (b) band connectivity matrices averaged across the 7 participants. Brain plots show the top 200 connections.

Results from the resting-state OPM-MEG connectivity study are shown in Fig. 5. Group-average connectivity matrices in the alpha (panel a) and beta (panel b) bands are plotted. The 3D brain plots show dominant connections between AAL regions (200 connections with the highest connectivity value). Differences between alpha- and beta-band connectomes are clear; alpha oscillations support connections between occipital and motor regions (with some frontal projections), whilst the beta-band connectome appears dominated by sensorimotor and fronto-parietal connectivity.

**Fig. 6.**
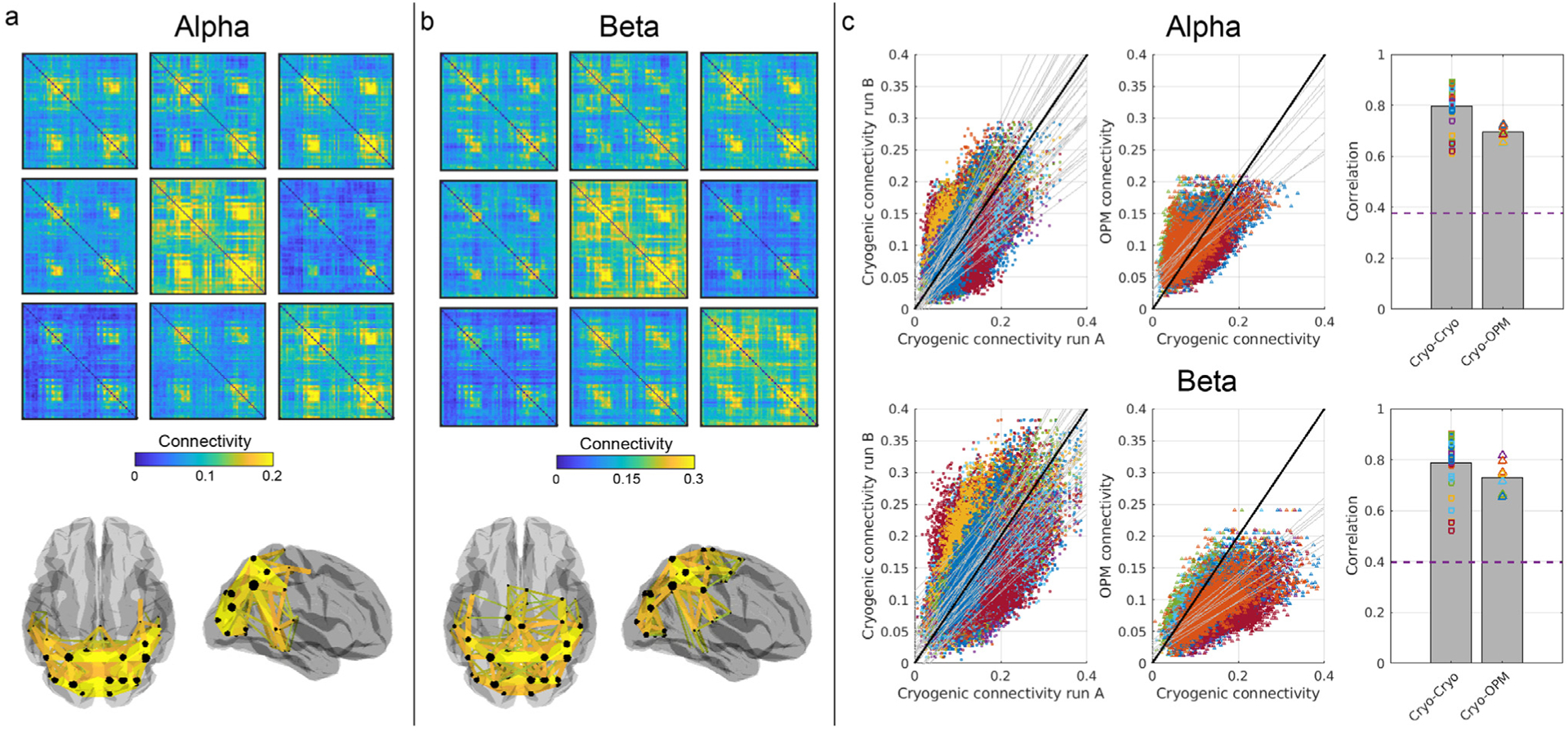
Resting-state group connectivity matrices from cryogenic data and a comparison with the OPM-derived connectome. Alpha- (a) and beta- (b) band connectivity matrices from 9 groups of 7 subjects. 3D brain plots show dominant connections (top 200). Note that even though these are group-averaged results, clear differences across groups remain (although the overall pattern appears robust). c) Results for alpha (top row) and beta (bottom row). The scatter plots on the left show cryogenic-derived connectivity values, with different groups plotted against each other i.e. each data point shows connectivity for the same connection, in two different subject groups, plotted against each other. The black line shows *y* = *x*; the grey lines show lines of best fit for the 36 different possible comparisons between independent groups. The scatter plots in the centre show cryogenic-derived connectivity versus OPM-derived connectivity values. 9 separate comparisons are made between the OPM-derived connectome (averaged across 7 subjects) and 9 separate cryogenic-derived connectomes (each the average of 7 subjects). The bar chart shows mean correlation values for cryogenic-to-cryogenic connectivity (left-hand bar) and OPM-to-cryogenic connectivity (right-hand bar). The individual points (squares/triangles) show individual correlation values from all possible matrix parings. The dashed line shows the 99th percentile of the null distribution.

Resting-state connectivity results, derived from cryogenic data, are plotted in Fig. 6. Panels a and b show alpha- and beta-band connectivity matrices, respectively. In each panel, 9 different matrices are shown: these correspond to the 9 (randomly selected) groups of 7 subjects. Colour bars are the same for all matrices. The 3D brains show the dominant connections (again the 200 connections with the highest connectivity values; these are derived from the grand average across 63 subjects). Here we see that alpha oscillations mediate connections primarily in occipital areas whilst the beta-band connectome shows a more widespread connectivity, between occipital and parietal regions. Interestingly, whilst a common structure exists across all groups, in both bands, there is large discrepancy between connectivity strength values across groups.

Fig. 6c shows a comparison between cryogenic- and OPM-derived resting-state connectomes. The upper and lower rows show results for alpha and beta bands, respectively. In the scatter plots, we show resting-state connectivity values from different groups of people, plotted against each other. In the left-hand scatter plot, 36 different comparisons are made, corresponding to 36 available pairs within our 9-group connectivity matrices from cryogenic data. The black line shows ‘*y* = *x*’ and, given that the points represent connectivity values derived using the same system, for the same connections, in different subject groups, we would expect to see a scatter around this line. This is broadly the case, however it is important to note how wide the variation around this line is. This reflects differences between subjects/groups. The middle scatter plot contains 9 comparisons. Here, our group-averaged OPM-derived connectivity values are plotted against equivalent values for each of the 9 cryogenic group averages. Note here that for both the alpha and beta band, although data do not necessarily lie along the ‘*y* = *x*’ line, a very clear linear trend is observed suggesting that, in general, the OPM and cryogenic resting-state connectomes are well matched. As with the task-based connectivity, we found that in the beta band, connectivity values across the matrix were lower for OPMs than for the cryogenic system. Specifically, the mean (across the connectome matrix) connectivity value was 0.09 ± 0.03 (OPM) compared to 0.14 ± 0.06 (cryogenic) (average ± standard deviation across subjects). However, this was not the case for the alpha band where OPM-derived connectivity was slightly higher (0.10 ± 0.04 (OPM) compared to 0.09 ± 0.04 (cryogenic)).

Finally, the right-hand bar chart shows correlation values between group-level connectomes; the left-hand bar shows cryogenic vs cryogenic connectomes (36 separate comparisons between the 9 group connectivity matrices); the right-hand bar shows OPM vs cryogenic connectomes (9 comparisons between the mean OPM connectome and the 9 cryogenic groups). The bars show averages, whilst all data contributing to those averages are shown overlaid as squares/triangles. The dashed line corresponds to the 99th percentile of the null distribution; that is, the cryogenic-to-OPM correlation should cross this threshold to be above chance. Here we see a clear result. Firstly, there is clearly a significant correlation between OPM- and cryogenic-derived connectivity matrices. Second, whilst the average OPM-to-cryogenic correlation (0.68 for alpha and 0.74 for beta) is marginally lower than cryogenic-to-cryogenic comparisons (0.80 for both alpha and beta) (probably a result of coverage bias – see Discussion) the range of OPM-to-cryogenic correlation values is well contained within the range of cryogenic-to-cryogenic values suggesting no measurable difference between the two. Consequently, we conclude that OPM and cryogenic connectivity are approximately equivalent.

## 4. Discussion

OPMs represent a step change for MEG instrumentation: OPM-MEG offers the potential for cheaper MEG systems which can ultimately come into more widespread use, particularly in clinical settings. Wearable helmets mean that sensors move with the head, removing worries around subject movement which can lead to data becoming unusable in cryogenic systems (Boto et al., 2018). Flexible placement of small and lightweight sensors means that, in principle, an OPM-MEG system can adapt to any head shape or size (Hill et al., 2020). Ultimately this means that OPM-based systems are better able to accommodate challenging patient groups, in particular children (with smaller heads) or subject groups who find it hard to keep sufficiently still in a conventional scanning environment. The ability to move whilst scanning opens up new possibilities for neuroscientific experimentation – for example we can scan people as they undertake naturalistic tasks (Hill et al., 2019) or become immersed in a virtual environment (Roberts et al., 2019). Finally, because OPM sensors can get closer to the brain, we can capture better data with higher sensitivity and spatial resolution (Boto et al., 2019, 2016; Iivanainen et al., 2017). These factors point towards OPMs superseding cryogenic MEG devices in the coming years. However, the technology remains largely unproven, and it is critical that OPM-MEG systems begin to demonstrate that they can perform as well as (or preferably better than) cryogenic systems for neuroscientific measurements.

Functional connectivity is an area that has become of great importance in recent years. Canonical networks, and the functional connectivities that underlie them, are fundamental to healthy brain function and have been shown to be perturbed in a number of abnormalities ranging from mental health disorders that strike in the very young, to neurodegenerative conditions that become a problem for the elderly. The combination of high spatial and temporal resolution makes MEG, arguably, the technique of choice for measurement of brain network activity and connectivity. This is particularly true for the measurement of dynamic connectivity (e.g. during a task) where we might aim to probe the formation and dissolution of transient networks as they modulate to support cognition. It is for these reasons that functional connectivity and network measurement represent a key part of MEG research. Consequently, showing that OPM-MEG systems are capable of such measurements, with similar fidelity to conventional devices, is a key step forward in the journey towards a viable OPM-MEG device.

Here, we aimed to show that OPM-MEG could offer characterisation of the brain-wide functional connectome. As noted in our introduction, such demonstration relies not only on high fidelity (unaveraged) MEG data, but also on whole-brain coverage, spatial specificity and the reconstruction of large numbers of sources; given the limited number of channels in OPM-MEG systems (~50 compared to ~300 in cryogenic systems) these latter points could have posed a challenge. However, results show that 50-channel OPM-MEG, in combination with accurate co-registration procedures and an appropriate source localisation algorithm, can measure functional connectivity with similar efficacy to a cryogenic system. At face value, this is perhaps surprising, but we note that previous electroencephalography (EEG) studies have shown that connectivity patterns can be measured using relatively small numbers of sensors. For example, Siems et al. (2016) directly compared connectivity measures using 275-channel (cryogenic) MEG and 64-channel EEG systems. Results showed that similar brain networks are observable (albeit with less spatial specificity in EEG – likely a consequence of the EEG forward model, which is complicated by inhomogeneous conductivity across the brain skull and scalp). The likely reason that fewer channels results in similar data is field spread: because the magnetic field from a single source affects multiple sensors, there is a degree of redundancy across an array of MEG sensors. This means that at least some signal from each of the 78 AAL regions will be captured by one or more OPMs, even with a relatively low channel count. However, we also stress that, because OPMs get closer to the head, the field topography at the scalp is less diffuse compared to cryogenic systems and so channel redundancy is diminished. Further, it is known (Boto et al., 2016; Iivanainen et al., 2017) that the performance of any MEG system improves as sensors are added – there are three reasons for this: first, extra sensors enable capture of higher spatial frequencies in the scalp topography, which will improve spatial resolution. Second, extra sensors enable us to resolve more sources in the brain (i.e. they will reduce source leakage). Third, extra sensors enable higher SNR, partly because there are more measurements over which the signal can be averaged and partly because the array is better able to characterise interference (which can then be supressed). Consequently, whilst 50-channel OPM-MEG offers good characterisation of the human connectome, the addition of more channels will always provide a significant advantage. OPM-systems with e.g. hundreds of channels should therefore remain an ambition.

Our task-based connectivity demonstration showed that both cryogenic- and OPM-MEG yield robust networks in the alpha, beta and gamma bands in response to a visuo-motor paradigm. Of interest here is the ability to measure individual differences between subjects. We showed that, in the beta band, both systems measured a robust sensorimotor network which exhibited significantly more bilateral connectivity in subject two, compared to subject 1. Of course, the reason for these differences between subjects is unclear, but the fact that the same differences were observed using both cryogenic and OPM-MEG is compelling. We argue that this finding is important for two reasons. First, from an OPM-MEG point of view, it validates the fact that the MEG data are of high quality; indeed the ability to “tell which subject you are scanning” based only on the MEG data is a satisfying demonstration of the equivalence between OPM- and cryogenic-derived data. Second, more broadly, this finding demonstrates the importance of inter-individual differences. Many clinically-oriented MEG studies employ cross-sectional designs where large subject numbers from different ‘groups’ are scanned and differences between groups sought. However, here we show that sizeable differences between two healthy individuals can be robustly observed and it is tempting to speculate that these differences are larger than the more subtle deviations that are often observed between groups. It follows that, given the robustness of the within-subject measures, it is likely that acquisition of longitudinal datasets, tracking how e.g. a patient’s brain changes throughout the course of an illness, may ultimately be more fruitful (and more useful) than cross-sectional group studies. Finally, an important methodological point relating to task-based connectivity is that the analysis method used here (amplitude envelope correlation) may highlight connections that are not driven by intrinsic coupling *per se*. For example, two unrelated brain regions could both be modulated (independently) by the task and would appear “connected”. Here, our aim was to show similarity or differences between OPM and cryogenic MEG systems and with this in mind, we believe this caveat can be overlooked, however it should be taken into account in future studies of connectivity.

In many ways, our resting-state data posed a greater challenge for OPM-MEG compared to task-based data, for the simple reason that the task-based connectome could be averaged over trials, potentially masking the effect of any artefacts at the sensor level. Conversely, resting-state connectivity must be inferred based on unaveraged data, meaning that sensor artefacts could have a greater influence. Our findings showed that similar resting-state network structure could be elucidated both using cryogenic and OPM-based MEG. Our beta-band analyses (taken from Fig. 6c) showed that, when considering a group result across 7 subjects, cryogenic-derived connectome matrices showed 80% correlation; when comparing OPM- and cryogenic-derived connectomes, this was reduced marginally to 74%. In the alpha band, this reduction was somewhat larger with 80% correlation for cryogenic-derived connectomes reducing to 68% for OPM-cryogenic comparison. These reductions are not surprising considering the vast differences between the systems – in particular, the channel count and differences in spatial coverage (see also below). However, in both cases these reductions were small compared to the range of possible correlation values and could easily be due to differences in the groups of participants scanned. These data therefore show that OPM-MEG, even with a modest number of sensors, is able to effectively reproduce the human connectome measurable by cryogenic MEG.

An important consideration for connectivity measurement is data quality. Given the fact that OPMs are configured as magnetometers, as distinct from gradiometers, we might expect a higher degree of interference in our OPM compared to our cryogenic data. In fact, further analysis (see Appendix) showed that magnetic artefacts of no interest are present in OPM-MEG sensor level data, however, using beamforming these artefacts are likely to be eliminated efficiently. This is an important point; at present, commercial grade OPMs are formed as magnetometers. Whilst it is possible to form gradiometers from two magnetometers, and such methods have been shown to be effective (Hill et al., 2019), this involves a digital subtraction of signals from two adjacent sensors which necessarily means (assuming a simple Gaussian model) a $\sqrt{2}$ increase in sensor noise. Recent work has shown that inherent OPM-gradiometers (i.e. OPMs where the same light is passed through two separate vapour cells, eliminating the need for digital subtraction and consequently the $\sqrt{2}$ noise increase) are possible to construct (Nardelli et al., 2020). However, axial gradiometers would require cells to be stacked on top of one another (i.e. radial to the head) and any reasonable (e.g. 5 cm) baseline would make a wearable helmet bulky and arguably impractical. In addition, planar gradiometers (two cells separated tangentially along the scalp surface) with a long baseline would limit the numbers of sensors that fit around the head; and shorter baseline planar gradiometers limit depth sensitivity. With this in mind, it is possible that wearable systems with high channel counts and gradiometer-based sensors may be challenging. Consequently, it is positive that mechanisms such as beamforming work well for reduction of interference. It remains to be seen as to whether other interference rejection strategies (for example signal source separation; (Taulu and Simola, 2006)) are also effective, but as shown in our Appendix, effective interference minimisation will be extremely important for future OPM-MEG studies.

Whilst OPM- and cryogenic-derived connectomes were largely similar, there are some differences which are worth noting. First, in the beta band, OPM-derived functional connectivity values were generally lower in magnitude than their cryogenic equivalents. At face value this could suggest increased noise in the OPM data which would diminish connectivity. However alpha connectivity values were marginally higher (in the resting state) for the OPM system (and comparable for the task-based data). In addition, we know from previous analyses on the same (task) data (Hill et al., 2020) that SNR values in the OPM and cryogenic data are approximately the same (in the beta band in motor cortex, source-localised SNR was 22 ± 4 in the OPM system compared to 21 ± 4 in the cryogenic system). We therefore think it unlikely that increased noise is responsible for the systematic connectivity reduction in OPMs. There are two potential more likely explanations. First, it is possible that reduced connectivity could result from the smaller number of channels: with only 50 channels in the OPM helmet (compared to 275 SQUID channels), theory would predict that there would be more spatial blurring across the 78 AAL regions, and consequently increased source leakage. This leakage would be addressed by our leakage reduction algorithm (pairwise orthogonalisation) with the likely result being diminished connectivity values; this potentially suggests that a general reduction in connectivity with lower channel count is possible. Second, lower overall (average) connectivity could result from changing spatial coverage; in particular, if the OPM system was less sensitive, and the cryogenic system proportionally more sensitive, to regions which demonstrate high functional connectivity.

Comparing Figs. 5 and 6 we see that, for the resting state, even though connectomes are largely similar (evidenced by the high correlation values) the 200 connections with the highest connectivity show a different spatial pattern in OPM compared to cryogenic MEG; specifically, for the cryogenic data, in both the alpha and beta bands, connectivity is largest in the occipital and parietal lobes whereas for OPM data, the alpha band is more widespread, including frontal regions while for beta, the motor system and fronto-parietal connections are highlighted. We believe that these relatively large differences are due to coverage. Sensitivity in the cryogenic system is greatest at the back of the head; this is a known problem because subjects tend to sit with their head resting on the rear of the cryogenic helmet, meaning high SNR for visual/parietal areas and poor coverage in frontal lobes. Indeed this was shown by sensitivity plots in Hill et al. (2020) and is supported by a recent study (Coquelet et al. (2020)) which showed that EEG yields higher frontal connectivity compared to (conventional) MEG; the authors cited an increased gap between the MEG sensors and the brain, in frontal regions, as the reason. Conversely, the additively-manufactured OPM helmet used in this study was a good fit for most subjects over the top of the head, but a noticeable gap (of ~1–2 cm) exists across the back of the head. This gap acts to move sensors away from occipital lobe and, consequently, signals will diminish, as will connectivity. It is possible that diminishing the naturally high connectivity values in beta band in visual cortex could have contributed towards the overall lower values of connectivity observed in the OPM system compared to the cryogenic system. Likewise, because the OPM system brings sensors closer to frontal cortices, this could explain the increased sensitivity to frontal alpha connectivity in OPMs. In general, it is clear that the OPM helmet, despite problems over the occipital lobe, has more uniform coverage than cryogenic MEG. This likely means that for cryogenic MEG the spatial signature of the connectome matrix rides on top of a sensitivity profile which diminishes frontal lobe contributions. The upshot would be that when measuring repeatability (i.e. the correlation measures used in Figs. 3 and 6) underlying coverage-based modulation would inflate correlation for cryogenic-to-cryogenic comparison, possibly explaining (in part) the results shown. What is clear is that inhomogeneous brain-to-sensor spacing for different areas of cortex can have a marked effect on connectivity results and this must be taken into account in future generations of scanner design. One potential solution is to use individualised scanner-casts, however these tend to be difficult and time-consuming to generate and are expensive. The introduction of more sophisticated helmets that allow a degree of adaptation to different head shapes (e.g. by including built-in facility to adjust sensor positions along the radial direction), could negate this problem.

Finally, it is important to note the current state of maturity of OPM-MEG technology. At the time of writing, to our knowledge, all operational OPM-MEG systems are “home-made”; that is, constructed by research groups “in house” based on OPM sensors, magnetic shielding, coils for field control and bespoke integrative data acquisition and control electronics, and software. Commercial grade OPMs, appropriate for MEG applications, are now available from at least two vendors (www.quspin.com; www.fieldlineinc.com). Further, OPM-optimised magnetic shielding, including electromagnetic coils similar to those used here, are also now available commercially (www.magneticshields.co.uk). However, to date there is no commercial solution for an integrated system. That said, it is clear from the results presented here and in other recent demonstrations that OPM technology is progressing rapidly and can now compete with cryogenic MEG technology. Furthermore, recent results show clearly the advantages of OPM-MEG compared to the widely established EEG (vastly improved spatial resolution and tenfold reduced sensitivity to non-neural signals (e.g. from muscles; (Boto et al., 2019)). It therefore seems highly likely that commercial integrated OPM-MEG systems will become available in the near future. Such systems have the potential to overtake cryogenic MEG systems, and even possibly replace some EEG systems for clinical evaluation of patients with neurological problems such as epilepsy.

## 5. Conclusion

In conclusion, our study has shown that OPM-MEG can measure whole-brain functional connectivity with a fidelity similar to that demonstrated by conventional cryogenic MEG machines. In the resting state, our results show that connectome matrices from OPM and cryogenic systems exhibit an extremely high degree of similarity, with correlation values *>*70%. This value is not measurably different to the correlation observed between connectomes measured across different subject groups on a single cryogenic MEG device. In a task-based study, we showed that robust differences in connectivity between individuals (scanned multiple times) exist, and similar individualised features could be identified in cryogenic and OPM-MEG measurements, again demonstrating the fidelity of OPM-MEG data. OPMs offer a step change for MEG instrumentation, however OPM-MEG remains a nascent technology with significant work still to be done. The present demonstration takes us one step closer to routine use of OPM-MEG for neuroscientific measurement. This adds weight to the argument that OPMs will ultimately supersede cryogenic-based instrumentation.

## Supplementary Material

Appendix B

## Figures and Tables

**Fig. A1.**
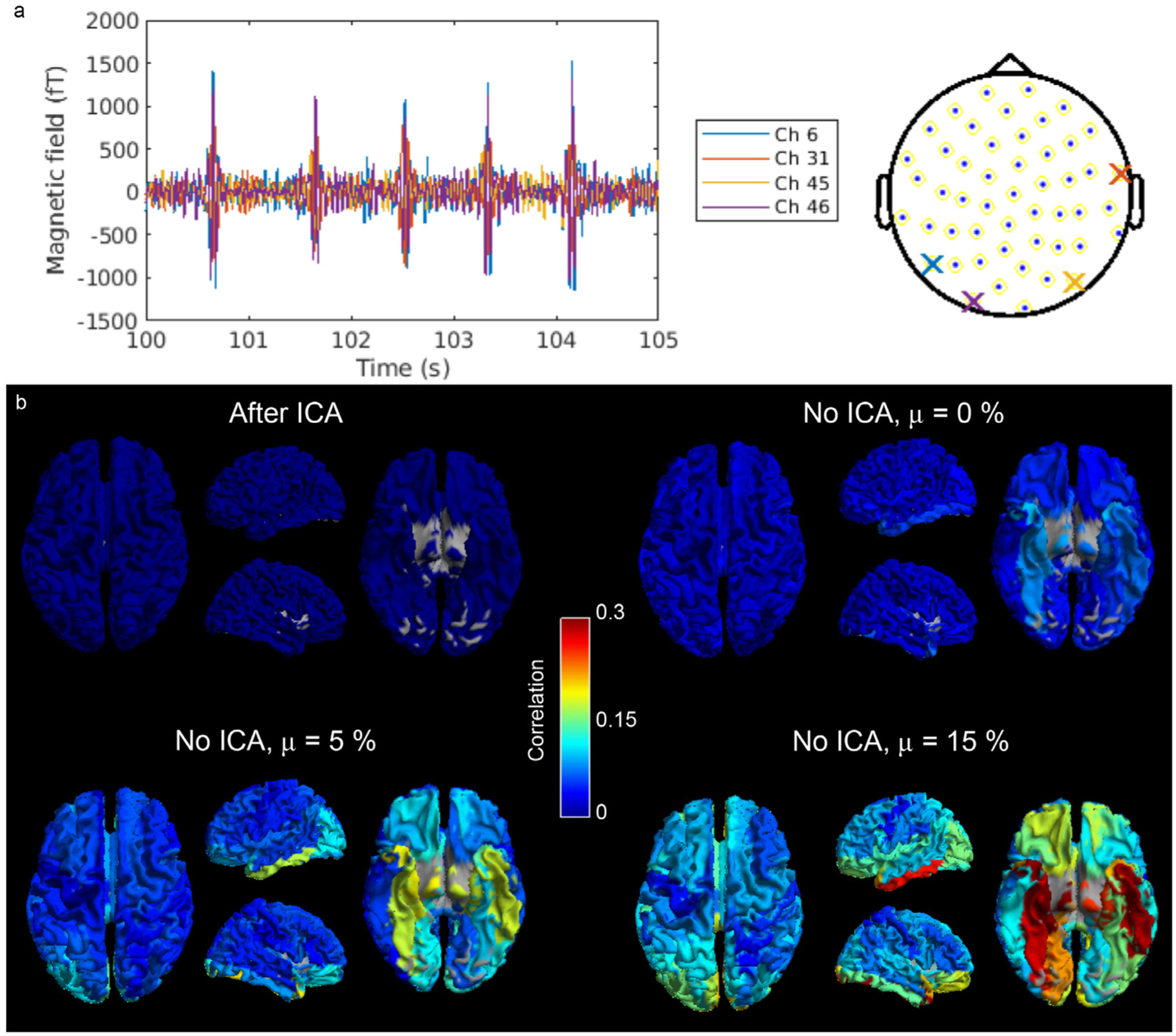
Presentation of the heartbeat artefact in OPM-MEG: a) Sensor-space data filtered to the beta band. Four channels are shown (indicated on the sensor layout on the right) and despite the separation of approximately 40 cm between the heart itself and the head-mounted sensors, the magnetocardiogram can be seen clearly. b) Correlation between the heartbeat artefact and source-localised data, across 78 AAL regions. Top left: beamforming applied with the cardiac artefact removed. Top right: beamforming applied to the full dataset with no regularisation. Bottom left: beamforming applied with 5% regularisation. Bottom right: beamforming applied with 15% regularisation.

## Appendix. External interference and its influence on OPM data

A major concern related to connectivity measurement using OPM-MEG is the influence of external magnetic interference. As outlined in our introduction, most cryogenic MEG systems employ gradiometers to reduce the effect of external magnetic fields. Some also use reference arrays, higher-order synthetic gradiometry (Vrba and Robinson, 2001), or software approaches (Taulu and Simola, 2006). However, OPMs are inherently formed as magnetometers and this means that interference is more problematic. This is a particular concern for connectivity measures (in the resting state) since averaging across trials is impossible. Thus, understanding how OPM data is influenced by interference is important.

A good example of interference from a distal source is the magnetic artefact from the heart. Even at a distance, the field from the heart is many times larger than the field from the brain. Also, the fundamental frequencies of the heartbeat overlap with the frequency bands of interest for connectivity (i.e. the alpha and beta ranges). Consequently, this poses a significant challenge since, if the heartbeat artefact appears across many sensors, and is projected (via source localisation) into brain space, this will necessarily inflate functional connectivity measurement. For these reasons, we aimed to test the extent to which the heartbeat artefact is found in source-localised OPM-MEG data.

### Methods

In order to probe the presence of a heartbeat artefact in OPM-MEG resting-state data, independent component analysis (ICA) was applied to beta-band filtered sensor-level data. ICA was applied in the temporal dimension using the fastICA algorithm (Hyvärinen, 1999); we selected a sufficient number of independent components to explain 95% of the data variance. This procedure was applied to data from all 7 subjects independently. For one subject, a clear heartbeat artefact was contained within a single independent component. For the other 6 subjects, the heartbeat was split across two independent components.

Following this, for all subjects we re-ran the beamformer spatial filter in order to reconstruct source-space data at the 78 pre-selected AAL regions. This was done in two different ways:

1. Following ICA, data were reconstructed with the heartbeat artefact removed (by removing either 1 or 2 independent components). The data covariance matrix was derived based on these reduced data, and a beamformer applied. Note that component removal in this way necessitates the application of matrix regularisation and so this was applied, using the Tikhonov method, with a regularisation parameter equal to 0.1% of the maximum singular value of the unregularised matrix.
2. Beamforming was applied without removal of the heartbeat artefact. This was done with no regularisation (as in our main manuscript) as well as with regularisation parameters equal to 5% and 15% of the maximum singular value of the unregularised matrix. (Note, the addition of regularisation reduces the ability of the beamformer to supress external magnetic interference, and consequently this proves a useful marker of how other source localisation algorithms (e.g. a dipole fit) might behave.)

In both cases we correlated the ICA-derived heartbeat to the source-localised MEG data in order to assess the influence of interference.

### Results and discussion

Fig. A1a shows representative beta-band filtered OPM-MEG data at the channel level. Data for four channels, for a single subject, are shown and we see that the beta-band component of the heartbeat is easily identified. Fig. A1b shows the cortical topography of correlation with the heartbeat artefact following source localisation. In the top left, we show the case where the heartbeat artefact was removed. The top right, bottom left, and bottom right show the case for a beamformer with zero, 5% and 15% regularisation, respectively. As expected, heartbeat correlation is zero in the case where the heartbeat has been removed (a simple consequence of ICA). More importantly, correlation is very close to zero (0.03 ± 0.02 (mean ± standard deviation across all 78 regions)) when an unregularised beamformer is applied. As regularisation is increased, correlation increases markedly (to 0.06 ± 0.04 and 0.11 ± 0.06 for 5% and 15% regularisation, respectively). Note that correlation is most pronounced for deeper regions.

It is clear from this result that the good performance of OPM-MEG in connectivity assessment observed in our manuscript is due, at least in part, to the noise rejection characteristics of the beamformer. In cases where beamforming is less efficacious, artefacts begin to leak into source-space data and as a consequence, connectivity measurement (or indeed any assessment of neural oscillatory processes) would become contaminated. However with a well-functioning beamformer, unregularised, such artefacts are well rejected. (An important point here is that beamforming becomes more accurate for longer data recordings (note that our OPM recordings were 7 min compared to the “standard” 5 min that is often employed). This will have helped with interference rejection.) Of course, the artefact as defined here can also be removed by other techniques (e.g. ICA – as demonstrated) but this relies on *a-priori* artefact identification. This was easy for the temporally well-characterised heartbeat, but is harder for less well-known sources of magnetic interference. On the other hand, the beamformer does not rely on *a-priori* assumptions. Further, if it works well on the heartbeat, we can assume it works equally well in nulling other sources of external magnetic interference. In conclusion, we recommend that beamforming (or at least an adaptive source-localisation technique with good interference rejection properties) is used when attempting to assess source-space functional connectivity using OPM-MEG. However it should be stressed that beamforming comes with specific caveats – in particular that spatially-separate but temporally-correlated sources will be supressed in source reconstructions; this has the potential (Sjøgård et al., 2019) to supress genuine functional connectivity.

## References

[R1] Altarev I, Fierlinger P, Lins T, Marino MG, Nießen B, Petzoldt G, Reisner M, Stuiber S, Sturm M, Taggart Singh J, Taubenheim B, Rohrer HK, Schläpfer U, 2015. Minimizing magnetic fields for precision experiments. J. Appl. Phys 117. doi:10.1063/1.4922671.

[R2] Baker AP, Brookes MJ, Rezek IA, Smith SM, Behrens T, Smith PJP, Woolrich M, 2014. Fast transient networks in spontaneous human brain activity. Elife 2014, e01867. doi:10.7554/eLife.01867. PMC3965210 24668169

[R3] Barry DN, Tierney TM, Holmes N, Boto E, Roberts G, Leggett J, Bowtell R, Brookes MJ, Barnes GR, Maguire EA, 2019. Imaging the human hippocampus with optically-pumped magnetoencephalography. Neuroimage 203. doi:10.1016/j.neuroimage.2019.116192. PMC6854457 31521823

[R4] Beckmann CF, DeLuca M, Devlin JT, Smith SM, 2005. Investigations into resting-state connectivity using independent component analysis. Philos. Trans. R. Soc. B Biol. Sci 360, 1001–1013. doi:10.1098/rstb.2005.1634. PMC1854918 16087444

[R5] Benjamini Y, Hochberg Y, 1995. Controlling the false discovery rate: a practical and powerful approach to multiple testing. J. R. Stat. Soc. Ser. B doi:10.1111/j.2517-6161.1995.tb02031.x.

[R6] Biswal B, Zerrin Yetkin F, Haughton VM, Hyde JS, 1995. Functional connectivity in the motor cortex of resting human brain using echo-planar MRI. Magn. Reson. Med 34, 537–541. doi:10.1002/mrm.1910340409. 8524021

[R7] Borna A, Carter TR, Colombo AP, Jau YY, McKay J, Weisend M, Taulu S, Stephen JM, Schwindt PDD, 2020. Non-invasive functional-brain-imaging with an OPM-based magnetoencephalography system. PLoS One 15. doi:10.1371/journal.pone.0227684. PMC6980641 31978102

[R8] Boto E, Bowtell R, Krüger P, Fromhold TM, Morris PG, Meyer SS, Barnes GR, Brookes MJ, 2016. On the potential of a new generation of magnetometers for MEG: a beamformer simulation study. PLoS One 11, e0157655. doi:10.1371/journal.pone.0157655. 27564416 PMC5001648

[R9] Boto E, Holmes N, Leggett J, Roberts G, Shah V, Meyer SS, Muñoz LD, Mullinger KJ, Tierney TM, Bestmann S, Barnes GR, Bowtell R, Brookes MJ, 2018. Moving magnetoencephalography towards real-world applications with a wearable system. Nature 555, 657–661. doi:10.1038/nature26147. 29562238 PMC6063354

[R10] Boto E, Holmes N, Tierney TM, Leggett J, Hill R, Mellor S, Roberts G, Barnes GR, Bowtell R, Brookes MJ, Boto E, Holmes N, Tierney TM, Leggett J, Hill R, Mellor S, Roberts G, Barnes GR, Bowtell R, Brookes MJ, 2020. Magnetoencephalography using optically pumped magnetometers. In: Fifty Years of Magnetoencephalography. Oxford University Press, New York, pp. 104–124. doi:10.1093/oso/9780190935689.003.0008.

[R11] Boto E, Meyer SS, Shah V, Alem O, Knappe S, Kruger P, Fromhold TM, Lim M, Glover PM, Morris PG, Bowtell R, Barnes GR, Brookes MJ, 2017. A new generation of magnetoencephalography: room temperature measurements using optically-pumped magnetometers. Neuroimage 149, 404–414. doi:10.1016/j.neuroimage.2017.01.034. 28131890 PMC5562927

[R12] Boto E, Seedat ZA, Holmes N, Leggett J, Hill RM, Roberts G, Shah V, Fromhold TM, Mullinger KJ, Tierney TM, Barnes GR, Bowtell R, Brookes MJ, 2019. Wearable neuroimaging: combining and contrasting magnetoencephalography and electroencephalography. Neuroimage 201. doi:10.1016/j.neuroimage.2019.116099. PMC8235152 31419612

[R13] Bright MG, Whittaker JR, Driver ID, Murphy K, 2020. Vascular physiology drives functional brain networks. Neuroimage 217. doi:10.1016/j.neuroimage.2020.116907. PMC7339138 32387624

[R14] Brookes MJ, Hale JR, Zumer JM, Stevenson CM, Francis ST, Barnes GR, Owen JP, Morris PG, Nagarajan SS, 2011a. Measuring functional connectivity using MEG: methodology and comparison with fcMRI. Neuroimage 56, 1082–1104. doi:10.1016/j.neuroimage.2011.02.054. 21352925 PMC3224862

[R15] Brookes MJ, Vrba J, Robinson SE, Stevenson CM, Peters AM, Barnes GR, Hillebrand A, Morris PG, 2008. Optimising experimental design for MEG beamformer imaging. Neuroimage 39, 1788–1802. doi:10.1016/j.neuroimage.2007.09.050. 18155612

[R16] Brookes MJ, Woolrich MW, Barnes GR, 2012. Measuring functional connectivity in MEG: a multivariate approach insensitive to linear source leakage. Neuroimage 63, 910–920. doi:10.1016/j.neuroimage.2012.03.048. 22484306 PMC3459100

[R17] Brookes MJ, Woolrich MW, Luckhoo H, Price D, Hale JR, Stephenson MC, Barnes GR, Smith SM, Morris PG, 2011b. Investigating the electrophysiological basis of resting state networks using magnetoencephalography. Proc. Natl. Acad. Sci 108, 16783–16788. doi:10.1073/pnas.1112685108. 21930901 PMC3189080

[R18] Cohen D, 1972. Magnetoencephalography: detection of the brain’s electrical activity with a superconducting magnetometer. Science (80-.) 175, 664–666. doi:10.1126/science.175.4022.664. 5009769

[R19] Coquelet N, De Tiège X, Destoky F, Roshchupkina L, Bourguignon M, Goldman S, Peigneux P, Wens V, 2020. Comparing MEG and high-density EEG for intrinsic functional connectivity mapping. Neuroimage 210. doi:10.1016/j.neuroimage.2020.116556. 31972279

[R20] Engel AK, Gerloff C, Hilgetag CC, Nolte G, 2013. Intrinsic coupling modes: multiscale interactions in ongoing brain activity. Neuron 80, 867–886. doi:10.1016/j.neuron.2013.09.038. 24267648

[R21] Fox MD, Raichle ME, 2007. Spontaneous fluctuations in brain activity observed with functional magnetic resonance imaging. Nat. Rev. Neurosci doi:10.1038/nrn2201. 17704812

[R22] Gross J, Kujala J, Hämäläinen MS, Timmermann L, Schnitzler A, Salmelin R, 2001. Dynamic imaging of coherent sources: studying neural interactions in the human brain. Proc. Natl. Acad. Sci 98, 694–699. doi:10.1073/pnas.98.2.694. 11209067 PMC14650

[R23] Hill RM, Boto E, Holmes N, Hartley C, Seedat ZA, Leggett J, Roberts G, Shah V, Tierney TM, Woolrich MW, Stagg CJ, Barnes GR, Bowtell RR, Slater R, Brookes MJ, 2019. A tool for functional brain imaging with lifespan compliance. Nat. Commun 10. doi:10.1038/s41467-019-12486-x. PMC6831615 31690797

[R24] Hill RM, Boto E, Rea M, Holmes N, Leggett J, Coles LA, Papastavrou M, Everton SK, Hunt BAE, Sims D, Osborne J, Shah V, Bowtell R, Brookes MJ, 2020. Multi-channel whole-head OPM-MEG: helmet design and a comparison with a conventional system. Neuroimage doi:10.1016/j.neuroimage.2020.116995. PMC8274815 32480036

[R25] Hillebrand A, Tewarie P, Van Dellen E, Yu M, Carbo EWS, Douw L, Gouw AA, Van Straaten ECW, Stam CJ, 2016. Direction of information flow in large-scale resting-state networks is frequency-dependent. Proc. Natl. Acad. Sci. U. S. A doi:10.1073/pnas.1515657113. PMC4833227 27001844

[R26] Hipp JF, Hawellek DJ, Corbetta M, Siegel M, Engel AK, 2012. Large-scale cortical correlation structure of spontaneous oscillatory activity. Nat. Neurosci 15, 884–890. doi:10.1038/nn.3101. 22561454 PMC3861400

[R27] Holmes N, Leggett J, Boto E, Roberts G, Hill RM, Tierney TM, Shah V, Barnes GR, Brookes MJ, Bowtell R, 2018. A bi-planar coil system for nulling background magnetic fields in scalp mounted magnetoencephalography. Neuroimage 181, 760–774. doi:10.1016/j.neuroimage.2018.07.028. 30031934 PMC6150951

[R28] Homölle S, Oostenveld R, 2019. Using a structured-light 3D scanner to improve EEG source modeling with more accurate electrode positions. J. Neurosci. Methods 326, 108378. doi:10.1016/j.jneumeth.2019.108378. 31376413

[R29] Hoogenboom N, Schoffelen JM, Oostenveld R, Parkes LM, Fries P, 2006. Localizing human visual gamma-band activity in frequency, time and space. Neuroimage doi:10.1016/j.neuroimage.2005.08.043. 16216533

[R30] Hunt BAE, Tewarie PK, Mougin OE, Geades N, Jones DK, Singh KD, Morris PG, Gowland PA, Brookes MJ, 2016. Relationships between cortical myeloar-chitecture and electrophysiological networks. Proc. Natl. Acad. Sci 113, 13510–13515. doi:10.1073/pnas.1608587113. 27830650 PMC5127325

[R31] Hutchison RM, Womelsdorf T, Allen EA, Bandettini PA, Calhoun VD, Corbetta M, Della Penna S, Duyn JH, Glover GH, Gonzalez-Castillo J, Handwerker DA, Keilholz S, Kiviniemi V, Leopold DA, de Pasquale F, Sporns O, Walter M, Chang C, 2013. Dynamic functional connectivity: promise, issues, and interpretations. Neuroimage 80, 360–378. doi:10.1016/j.neuroimage.2013.05.079. 23707587 PMC3807588

[R32] Hyvärinen A, 1999. Fast and robust fixed-point algorithms for independent component analysis. IEEE Trans. Neural Netw 10, 626–634. doi:10.1109/72.761722. 18252563

[R33] Iivanainen J, Stenroos M, Parkkonen L, 2017. Measuring MEG closer to the brain: performance of on-scalp sensor arrays. Neuroimage 147, 542–553. doi:10.1016/j.neuroimage.2016.12.048. 28007515 PMC5432137

[R34] Iivanainen J, Zetter R, Parkkonen L, 2019. Potential of On-Scalp MEG: Robust detection of Human Visual Gamma-Band Responses bioRxiv 602342 doi:10.1101/602342. PMC7267937 31571310

[R35] Johnson CN, Schwindt PDD, Weisend M, 2013. Multi-sensor magnetoencephalography with atomic magnetometers. Phys. Med. Biol 58, 6065–6077. doi:10.1088/0031-9155/58/17/6065. 23939051 PMC4030549

[R36] Kamada K, Sato D, Ito Y, Natsukawa H, Okano K, Mizutani N, Kobayashi T, 2015. Human magnetoencephalogram measurements using newly developed compact module of high-sensitivity atomic magnetometer. Jpn. J. Appl. Phys 54, 026601. doi:10.7567/JJAP.54.026601.

[R37] Kim K, Begus S, Xia H, Lee SK, Jazbinsek V, Trontelj Z, Romalis MV, 2014. Multi-channel atomic magnetometer for magnetoencephalography: a configuration study. Neuroimage 89, 143–151. doi:10.1016/j.neuroimage.2013.10.040. 24185014

[R38] Luckhoo H, Hale JR, Stokes MG, Nobre AC, Morris PG, Brookes MJ, Woolrich MW, 2012. Inferring task-related networks using independent component analysis in magnetoencephalography. Neuroimage 62, 530–541. doi:10.1016/j.neuroimage.2012.04.046. 22569064 PMC3387383

[R39] Menon V, 2011. Large-scale brain networks and psychopathology: a unifying triple network model. Trends Cognit. Sci doi:10.1016/j.tics.2011.08.003. 21908230

[R40] Nardelli NV, Perry AR, Krzyzewski SP, Knappe SA, 2020. A conformal array of microfabricated optically-pumped first-order gradiometers for magnetoencephalography. EPJ Quantum Technol. doi:10.1140/epjqt/s40507-020-00086-4.

[R41] O’Neill GC, Bauer M, Woolrich MW, Morris PG, Barnes GR, Brookes MJ, 2015. Dynamic recruitment of resting state sub-networks. Neuroimage 115, 85–95. doi:10.1016/j.neuroimage.2015.04.030. 25899137 PMC4573462

[R42] Osborne J, Orton J, Alem O, Shah V, 2018. Fully integrated, standalone zero field optically pumped magnetometer for biomagnetism. Steep Dispers. Eng. Opto-At. Precis. Metrol XI, 10548.

[R43] Prichard D, Theiler J, 1994. Generating surrogate data for time series with several simultaneously measured variables. Phys. Rev. Lett doi:10.1103/PhysRevLett.73.951. 10057582

[R44] Raichle ME, 2009. A paradigm shift in functional brain imaging. J. Neurosci doi:10.1523/JNEUROSCI.4366-09.2009. PMC6665302 19828783

[R45] Roberts G, Holmes N, Alexander N, Boto E, Leggett J, Hill RM, Shah V, Rea M, Vaughan R, Maguire EA, Kessler K, Beebe S, Fromhold M, Barnes GR, Bowtell R, Brookes MJ, 2019. Towards OPM-MEG in a virtual reality environment. Neuroimage 199, 408–417. doi:10.1016/j.neuroimage.2019.06.010. 31173906 PMC8276767

[R46] Sander TH, Preusser J, Mhaskar R, Kitching J, Trahms L, Knappe S, 2012. Magnetoencephalography with a chip-scale atomic magnetometer. Biomed. Opt. Express 3, 981. doi:10.1364/BOE.3.000981. 22567591 PMC3342203

[R47] Schoffelen JM, Gross J, 2009. Source connectivity analysis with MEG and EEG. Hum. Brain Mapp 30, 1857–1865. doi:10.1002/hbm.20745. 19235884 PMC6870611

[R48] Seedat ZA, Quinn AJ, Vidaurre D, Liuzzi L, Gascoyne LE, Hunt BAE, O’Neill GC, Pakenham DO, Mullinger KJ, Morris PG, Woolrich MW, Brookes MJ, 2020. The role of transient spectral ‘bursts’ in functional connectivity: a magnetoencephalography study. Neuroimage 209. doi:10.1016/j.neuroimage.2020.116537. 31935517

[R49] Siems M, Pape AA, Hipp JF, Siegel M, 2016. Measuring the cortical correlation structure of spontaneous oscillatory activity with EEG and MEG. Neuroimage doi:10.1016/j.neuroimage.2016.01.055. 26827813

[R50] Sjøgård M, De Tiège X, Mary A, Peigneux P, Goldman S, Nagels G, van Schependom J, Quinn AJ, Woolrich MW, Wens V, 2019. Do the posterior mid-line cortices belong to the electrophysiological default-mode network? Neuroimage doi:10.1016/j.neuroimage.2019.06.052. 31238165

[R51] Smith SM, Fox PT, Miller KL, Glahn DC, Fox PM, Mackay CE, Filippini N, Watkins KE, Toro R, Laird AR, Beckmann CF, 2009. Correspondence of the brain’s functional architecture during activation and rest. Proc. Natl. Acad. Sci. U. S. A 106, 13040–13045. doi:10.1073/pnas.0905267106. 19620724 PMC2722273

[R52] Taulu S, Simola J, 2006. Spatiotemporal signal space separation method for rejecting nearby interference in MEG measurements. Phys. Med. Biol 51, 1759–1768. doi:10.1088/0031-9155/51/7/008. 16552102

[R53] Tewarie P, Bright MG, Hillebrand A, Robson SE, Gascoyne LE, Morris PG, Meier J, Van Mieghem P, Brookes MJ, 2016. Predicting haemodynamic networks using electrophysiology: the role of non-linear and cross-frequency interactions. Neuroimage doi:10.1016/j.neuroimage.2016.01.053. PMC4819720 26827811

[R54] Tierney TM, Holmes N, Mellor S, López JD, Roberts G, Hill RM, Boto E, Leggett J, Shah V, Brookes MJ, Bowtell R, Barnes GR, 2019. Optically pumped magnetometers: from quantum origins to multi-channel magnetoencephalography. Neuroimage doi:10.1016/j.neuroimage.2019.05.063, In Press. PMC6988110 31141737

[R55] Tierney TM, Holmes N, Meyer SS, Boto E, Roberts G, Leggett J, Buck S, Duque-Muñoz L, Litvak V, Bestmann S, Baldeweg T, Bowtell R, Brookes MJ, Barnes GR, 2018. Cognitive neuroscience using wearable magnetometer arrays: non-invasive assessment of language function. Neuroimage 181, 513–520. doi:10.1016/j.neuroimage.2018.07.035. 30016678 PMC6150946

[R56] Tzourio-Mazoyer N, Landeau B, Papathanassiou D, Crivello F, Etard O, Delcroix N, Mazoyer B, Joliot M, 2002. Automated anatomical labeling of activations in SPM using a macroscopic anatomical parcellation of the MNI MRI single-subject brain. Neuroimage 15, 273–289. doi:10.1006/nimg.2001.0978. 11771995

[R57] Vrba J, Robinson SE, 2001. Signal processing in magnetoencephalography. Methods 25, 249–271. doi:10.1006/meth.2001.1238. 11812209

[R58] Xia H, Ben-Amar Baranga A, Hoffman D, Romalis MV, 2006. Magnetoencephalography with an atomic magnetometer. Appl. Phys. Lett 89. doi:10.1063/1.2392722, Article number 211104.

[R59] Zetter R, Iivanainen J, Parkkonen L, 2019. Optical Co-registration of MRI and On-scalp MEG. Sci. Rep 9, 5490. doi:10.1038/s41598-019-41763-4. 30940844 PMC6445124

