# Supplementary material for: Measuring functional connectivity with wearable MEG
Source: Neuroimage. Author manuscript; Available in PMC 2021 Jun 21. (PMC8216250; doi:10.1016/j.neuroimage.2021.117815)
Supplement: Appendix B [file NIHMS1710574-supplement-Appendix_B.pdf]

Supplementary Material

Elena Boto, Ryan M. Hill, Molly Rea, Niall Holmes, Zelekha A. Seedat, James Leggett, Vishal Shah, James Osborne, Richard Bowtell[1] and Matthew J. Brookes

In the main manuscript, statistical significance of differences in connectivity strength between the two individuals (across 6 runs) was computed using a two-sample t-test. However, this technique assumes that data are normally distributed. Whilst this is a common assumption in MEG (indeed it is an assumption which underlies most source-localisation methodology) with such a small number of degrees of freedom (6) it is possible that the assumptions of normality are not met. For this reason, we also carried out a separate non-parametric test.

For each of the 78 AAL regions, a non-parametric Wilcoxon sum rank test was used to determine the statistical significance of differences between subjects. These calculations were performed for each scanner type separately (i.e. for a single scanner, and a single region, we tested whether the 6 connectivity strength measures from subject 1 were significantly different to the equivalent 6 connectivity strength measures from subject 2). Significance was assigned if the p-value was < 0.01. Figure S1 shows the result from this test: panel a is equivalent to that of Figure 4a, showing normalised connectivity strength for both participants and scanner type. Regions where subjects 1 and 2 were significantly different in both scanners are marked with yellow dashed lines and these same regions are shown on a 3D brain in panel b. Notice again that the regions highlighted (as for the t-test method) show higher interhemispheric connectivity in subject 2.

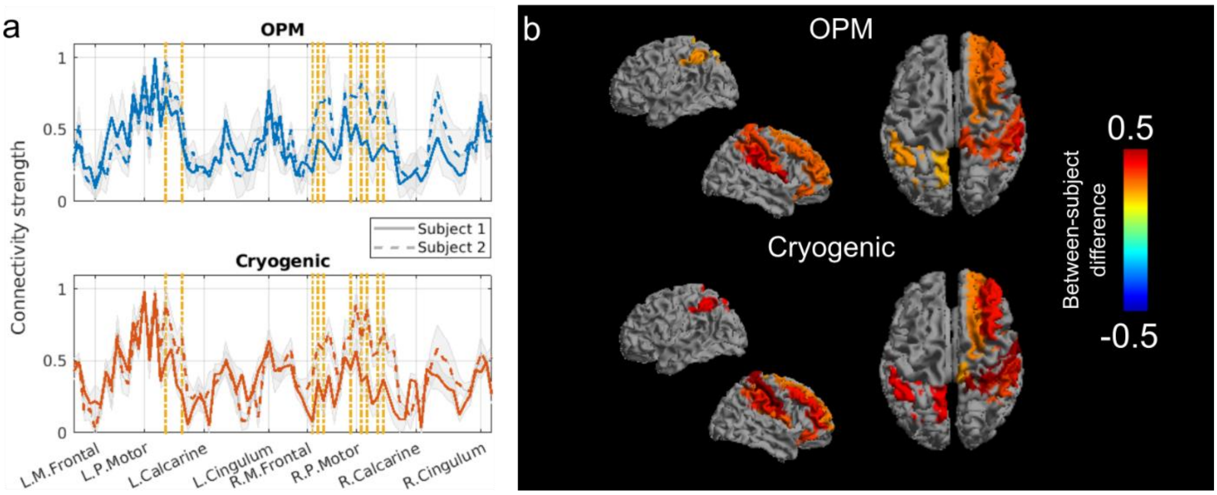

Figure S1: **Connectivity strength in the beta band.** a) Normalised connectivity strength recorded using cryogenic- (bottom) and OPM- (top) derived data for participants 1 (solid line) and 2 (dashed line). d) Brain areas showing significant difference between participants in both scanners (marked with yellow dashed lines in panel a).
